# Effect of Diurnal Fluctuating versus Constant Temperatures on Germination of 445 Species from the Eastern Tibet Plateau

**DOI:** 10.1371/journal.pone.0069364

**Published:** 2013-07-24

**Authors:** Kun Liu, Jerry M. Baskin, Carol C. Baskin, Haiyan Bu, Guozhen Du, Miaojun Ma

**Affiliations:** 1 State Key Laboratory of Glassland and Agroecosystems, School of Life Sciences, Lanzhou University, Lanzhou, P. R. China; 2 Department of Biology, University of Kentucky, Lexington, Kentucky, United States of America; 3 Department of Plant and Soil Sciences, University of Kentucky, Lexington, Kentucky, United States of America; Norwegian University of Science and Technology, Norway

## Abstract

Germination response to fluctuating temperatures is a mechanism by which seeds detect gaps in vegetation canopies and depth of burial in soil, and it is very important for plants. Thus, studies on the effect of fluctuating temperature on germination at the community level are valuable for understanding community structure and biodiversity maintenance. We determined the effects of two alternating temperatures (5/25°C and 10/20°C) and one constant temperature (15°C) on seed germination of 445 species in a grassland community on the eastern Tibet Plateau. Seed mass was determined for each species, and data on habitat, type of life cycle, altitudinal distribution and functional group (graminoids or forbs) were obtained from the literature. Taking all species into account, alternating temperatures increased germination percentages regardless of amplitude. Overall, species growing in disturbed ground showed a significant germination response to temperature fluctuation, but those living in Alpine/subalpine meadow, forest margin /scrub, marshland and dry sunny slope habitats did not. Species distributed only at high elevations (>2000m) did not show a significant germination response to temperature fluctuation, whereas those occurring at both high and low elevations had a significant positive response. Germination of annuals/biennials was significantly promoted by 5/25°C, but not by 10/20°C, whereas germination of perennials was significantly promoted by both 5/25°C and 10/20°C. Small-seeded species were more likely than large-seeded species to respond positively to fluctuating temperatures. Germination of forbs had a positive response to temperature fluctuation, but germination of graminoids did not. Regeneration ability by seeds for about 36% of the species studied in the grassland can be increased by temperature fluctuation. The differential response among species to alternating vs. constant temperatures helps maintain community structure and biodiversity. A positive germination response to temperature fluctuation can partly explain why there are more forbs in degraded meadows.

## Introduction

Germination is one of the most critical stages in the life cycle of plants [1,2], and it is the main source of variation in the regeneration niche [3], which is one of the core mechanisms maintaining biodiversity within a plant community. Thus, information on seed germination is required for understanding the distribution and life cycle traits of plant species and how they coexist in the same community. Germination is controlled by many environmental factors such as light, temperature and soil moisture [4–6]. These environmental factors can affect germination directly or indirectly. For example, temperature can regulate germination directly by activating enzymatic reactions occurring in the process of germination and by preventing or promoting the synthesis of hormones that affect the status of seed dormancy [5,7].

To a large extent, the abiotic factors required for seed germination can be described as the regeneration niche of a plant species. The temperature requirements for dormancy break and germination have been the focus of much research on seed ecology, and it has been shown that temperature plays a critical role in regulating seed dormancy-break and germination [5,8–12]. For example, if seeds require the low temperatures of winter for dormancy break, they usually become nondormant during winter and can germinate the following spring or early summer. On the other hand, if seeds require the high temperatures of summer for dormancy break, they usually become nondormant during summer and germinate in autumn. Minimum, maximum, optimum and fluctuating temperature requirements for seed germination can partly explain the germination niche of a species and thus its habitat requirements and distribution.

Although much attention has been given to the effects of temperature on seed dormancy break and germination, more work is needed to gain a better understanding of how temperature influences community structure through its effects on seed dormancy and germination. To correctly test this effect, the research should be done at the community level rather than at the individual or population level. However, we have not found any study on the effect of fluctuating temperatures on germination at the community level. Thus, we studied the effect of temperature fluctuation on seed germination of the species of a grassland community on the Tibet Plateau. We expected to obtain an integrated understanding of the effect of fluctuating temperatures on seed germination at the community level and to identify some adaptive mechanisms of species to high elevation on the Tibet Plateau.

In the field, maximum daily temperature fluctuation usually occurs at or near the soil surface in open habitats, and the daily amplitude of these fluctuations is decreased below a plant canopy [13,14] and with increasing depth of soil [15,16]. Thus, the positive response to temperature fluctuation is considered to be a mechanism that enables seeds to detect vegetation gaps and soil depth [17]. This mechanism ensures that germination occurs only in gaps and at/near the soil surface, thus avoiding death by shading and germinating deep in the soil. As such, then, temperature fluctuation as a seed germination cue is adaptive for plant regeneration by seeds.

Previous studies have indicated that seed germination behavior usually has some relationship with habitat, seed mass and type of life cycle [5,6,18]. For example, seed germination of most species in a wet habitat such as marshland usually is promoted by temperature fluctuation, whereas germination of woodland species usually does not respond positively to temperature fluctuation [19,20]. Fenner and Thompson argued that the germination of small-seeded species was more likely to be promoted by alternating temperatures than that of large-seeded species [6]. Bu et al. [18] found some differences in germination of annual and perennial species from the eastern Tibet Plateau; perennials usually germinated to higher percentages than annuals. There are more forbs in the degraded meadows (where there are lots of gaps) than in undisturbed meadows [21]. We predict that this difference may be related to the large temperature fluctuation near the soil surface of gaps in degraded meadows. In which case, the greater dependence of germination of forbs than graminoids on temperature fluctuation will restrict distribution of forbs in degraded meadows. Thus, determining the difference between germination response of forbs and graminoids to temperature fluctuation is important in testing the effects of temperature fluctuation on community structure. As thus then, habitat, seed mass, type of life cycle and functional group must be considered when studying the effect of temperature fluctuation on seed germination at the community level.

The atmosphere at high elevation is thin, resulting in high atmospheric transmissivity and strong solar irradiance [22]. Consequently, daytime temperatures on the Tibet Plateau are relatively high. However, the thin air cannot efficiently absorb thermal radiation emitted from the earth at night, and thus the temperature decreases sharply. As a result, there is a larger diurnal temperature fluctuation at high than at low elevations. A comparison of the germination responses of species restricted to high elevations with that of widespread species that grow at both high and low elevations to temperature fluctuation would enhance our understanding of the adaptations of plant species to the large diurnal temperature variations on the Tibet Plateau.

The purpose of the present study was to test the relationship between habitat, life cycle type, seed mass, altitudinal distribution and functional group and seed germination responses to temperature fluctuation of 445 species from the subalpine/alpine zone of the eastern Tibet Plateau in China, and give some implications for how decrease of diurnal temperature variation affect community structure by affecting seed germination. Thus, we evaluated the following two hypotheses(1). At the community level, temperature fluctuation can increase seed germination(2). Species that differ in seed mass, life cycle, habitat, altitudinal distribution or functional group differ in germination response to fluctuating temperature. This study provides a large amount of information on the germination responses of species in subalpine and alpine plant communities on the Tibet Plateau and thus enhances our understanding of their germination niches.

## Material and Methods

### Study site

The natural grasslands we studied are located on the eastern Tibet Plateau (101°–103° E, 34°–35.70° N). The altitude ranges from 2,800 to 4,200 m, and the climate is cold Humid-Alpine with a mean annual precipitation (snow and rainfall) of 450–780 mm. Mean annual temperature is 2-3 °C, and mean January and July temperatures are -10.7 and 11.7°C, respectively. There is an average of 270 frost days per year. The grassland type belongs mainly to alpine meadow, which is dominated by native monocots such as species of Poaceae and Cyperaceae and by native eudicots such as species of Ranunculaceae, Polygonaceae, Saxifragaceae, Asteraceae, Scrophulariaceae, Gentianaceae and Fabaceae [23].

### Seed collecting, weighing and testing for viability and germination

Seeds were collected in July to October in 2005, from private grasslands after we got permission from the owners. We traveled throughout the study site many times over different routes to collect ripe seeds of as many species as possible so that our database would be large enough to represent the whole grassland community. For a single species, seeds were collected from one site but from more than 20 plants or from all the plants that could be found if there were less than 20 of them. For each species, seeds were collected at the beginning of their dispersal period, so all seeds collected were mature. Soon after collection, seeds were cleaned and air-dried. Then, they were stored dry and allowed to afterripen at room temperature (15-20°C) until late March 2006. For every species, three subsamples of 100 air-dried seeds were haphazardly selected and weighed, and average mass per seed was calculated.

Before testing germination, we tested the viability of seeds of each species using the triphenyltetrazolium chloride test (TTC) [24]. Only species with a seed viability of ≥99% were used in the germination experiments. Seed germination was tested in the laboratory, beginning in late March 2006, in incubators (Conviron E15 Growth Chamber, Controlled Environments Ltd., Winnipeg, Canada) at two alternating temperatures [25°C (12h)/5°C (12h), 20°C (12h)/10°C (12h)] and one constant temperature (15°C). At the study site, in the germination season (late March to late May in spring or late August to late October in autumn), the daily temperature range is about 5-25°C near the soil surface in large gaps and about 10-20°C near the soil surface under vegetation. Thus, the alternating temperatures 5/25°C and 10/20°C were selected to approximate daily temperature regimes in a large gap and under vegetation, respectively. The constant temperature 15 °C was selected as control treatment of the two alternating temperatures.

These temperatures regimes have the same average temperature (15 °C). Thus, we could determine the differences between germination of seeds tested at an alternating temperature with a large amplitude, an alternating temperature with a small amplitude and a constant temperature. For each species, there were three replicates of 50 haphazardly-selected seeds each, and seeds were incubated on two sheets of filter paper moistened with distilled water in Petri dishes (9 cm diameter) in darkness; relative humidity in the chambers was about 70%. The seeds were checked for germination daily, at which time they were exposed to light for a few minutes; thus, any light requirement by the seeds likely was fulfilled during these exposures [5]. Germinated seeds (radicle visible) were removed from the Petri dishes at each counting; water was added to the filter paper as needed. To control mold, when we checked germination we also checked for fungi. When any seeds in a Petri dish were found to be attacked by fungi, we washed the seeds with distilled water and then put them in a new Petri dish. The germination tests lasted for 60 days.

### Data analyses

One of the main aims of our research was to determine the effect of amplitude of temperature fluctuation on community structure through its effect on seed germination at the community level. Thus, the main analysis contained all the species collected from the community we studied even though some of these species did not germinate well under any of the conditions at which they were tested. The normality of the data was not so good, thus, we analysed the data using nonparametric tests instead of ANOVA.

To test the effects of plant traits and environmental factors on germination response to temperature fluctuation, species were classified with respect to seed mass, life cycle type, habitat, altitudinal distribution and functional group, which were obtained from the China Flora Editing Group [25] (Table S1). Species were classed into two categories based on life cycle type: annuals/biennials and perennials. Seeds were sorted by mass into three size classes: 0-1, 1-2 and >2 mg (Table S2). Habitats were classified into five categories: alpine/subalpine meadow, disturbed ground, forest margin /scrub, marshland and dry sunny slope. According to the Flora of China [25], most lower limits of species occurring only on Tibet Plateau are ≥2000m. Thus, altitudinal distribution was divided into two classes: species distributed only above 2,000 m and those distributed both above and below 2000m. Functional groups were classified into two classes: graminoids and forbs. In the nonparametric tests, according to our experimental design (three temperature treatments on the same species combination, e.g. three observations on the same subject) we chose the Friedman Test (a nonparametric test for several related samples) with which to analyse the data. The Friedman test gave us a Post-hoc analysis, so three comparisons (10/20 vs. 15, 5/25 vs. 15 and 10/20 vs. 5/25) were made. Thus, we could know which groups are significantly different from each other. The effect of alternating temperature on germination of each group of species classified by habitat, seed mass, life cycle, altitudinal distribution or functional group was tested by the Friedman Test, so that we could know the effect of each of them on the germination response to alternating temperature.

## Results

**Figure 1:**
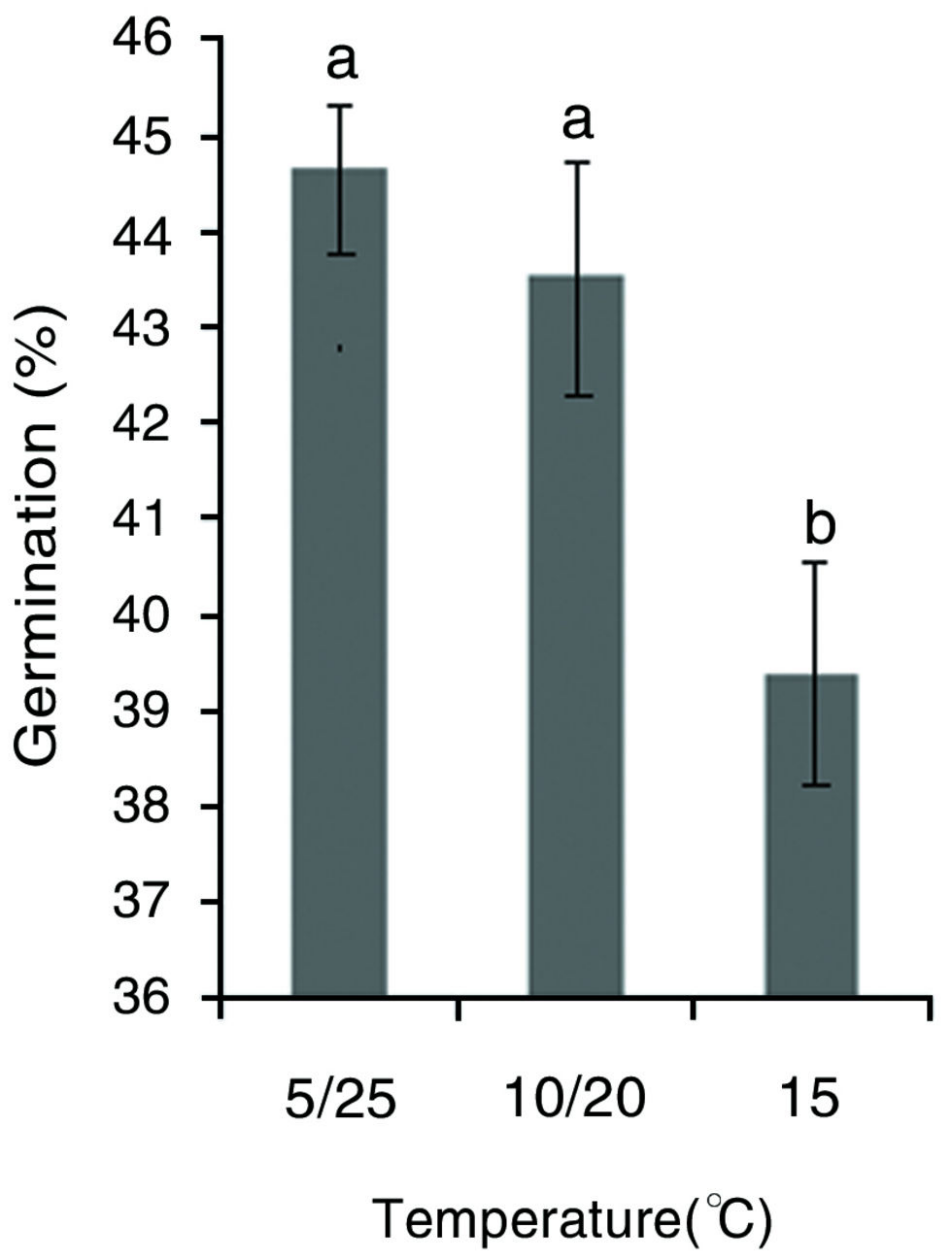
Effect of temperature fluctuation on germination percentage of 445 species from the eastern Tibet Plateau of China.

Alternating temperatures had a significant effect on mean germination percentage of the 445 species, which was significantly higher at the two alternating temperature regimes than it was at the one constant temperature (Figure 1). However, germination response varied with species and can be divided into four groups: I, 23.15% (103 of 445 species); seeds of these species had a negative germination response to temperature fluctuation; II, 35.96% (160 of 445 species); seeds of these species had a positive germination response to temperature fluctuation; III, 26.29% (117 of 445 species); seeds of these species did not germinate well regardless of the temperature fluctuation; and IV, 14.61% (65 of 445 species); seeds of these species attained a high germination percentage regardless of the magnitude of temperature fluctuation (Table S2).

### Effect of habitat on germination response to temperature fluctuation

**Figure 2:**
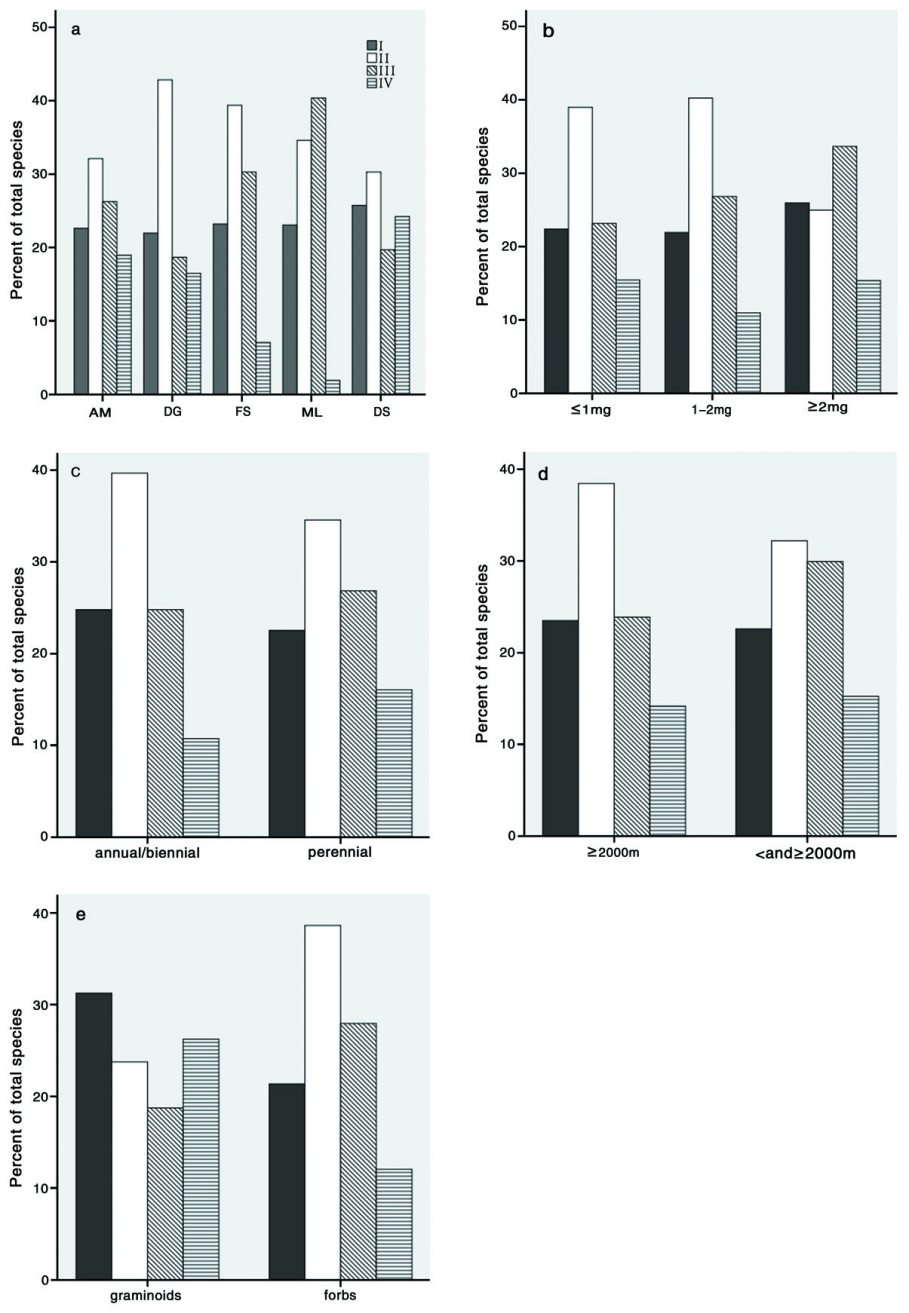
Relative importance of response groups I, II, III and IV when habitat (a), seed mass (b), life cycle type (c), altitudinal distribution (d) and functional group (e) are considered. I, species with a negative germination response to temperature fluctuation; II, species with a positive germination response to temperature fluctuation; III, species that cannot germinate well regardless of the temperature fluctuation; IV, species that can attain a high germination percentage regardless of the temperature fluctuation. AM: alpine/ subalpine meadow; DG: disturbed ground; FS: forest margin/scrub; ML: marshland; DS: dry sunny slope.

**Figure 3:**
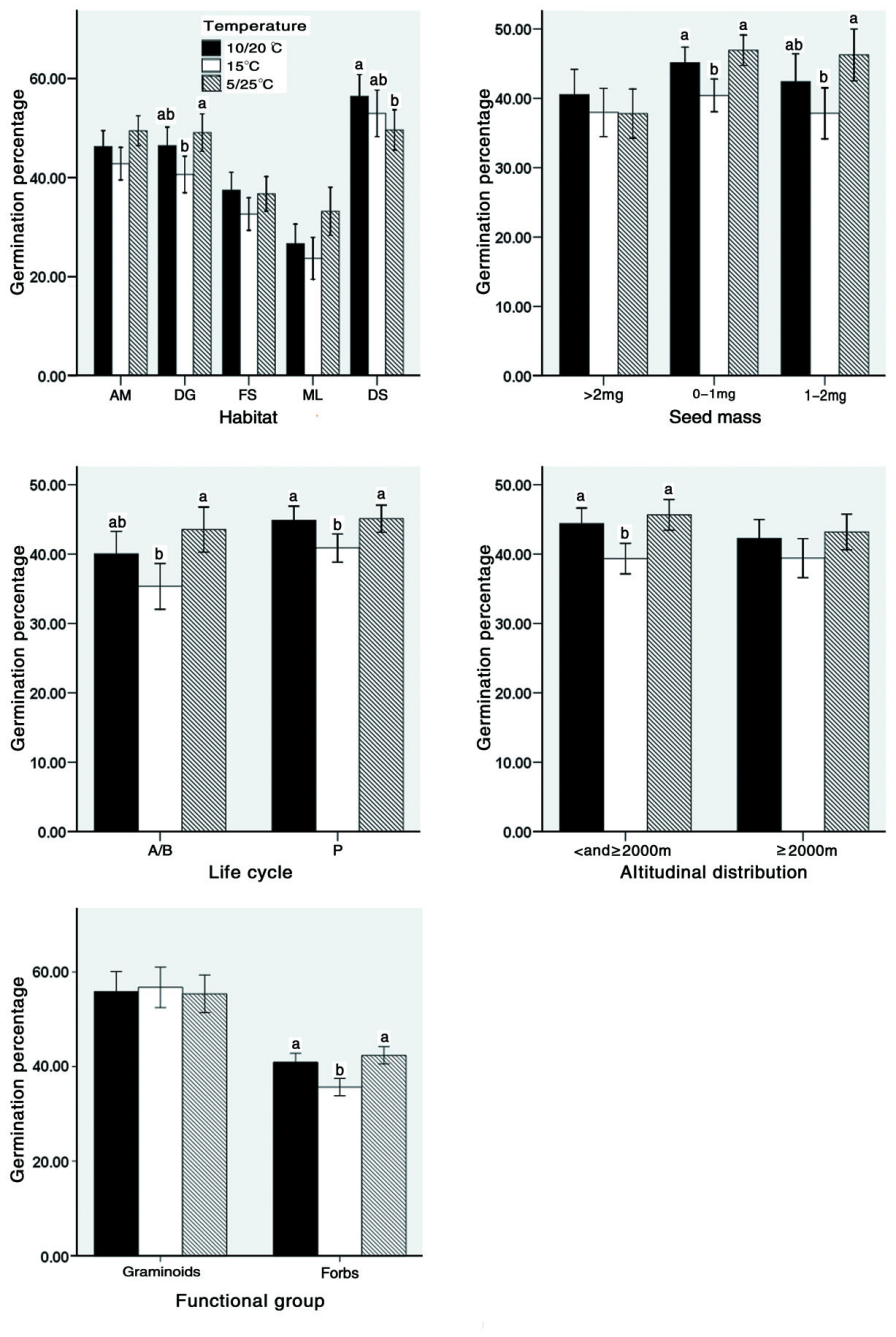
Germination percentages at different temperature regimes of species that differ in habitat, seed mass, life cycle type, altitudinal distribution and functional group. AM: alpine/ subalpine meadow; DG: disturbed ground; FS: forest margin/scrub; ML: marshland; DS: dry sunny slope; A/B: annuals/biennials; P: perennials; Error bars: ±SE. For each group of species, different letters on the bars indicate significant differences in germination under different temperature conditions at *P* < 0.05 (multiple comparisons with the Friedman Test).

Based on habitat, the order of proportion of species that responded positively to temperature fluctuation was disturbed ground > forest margin/scrub > marshland > alpine/subalpine meadow >dry sunny slope (Figure 2a). Our results (Table 1 Figure 3) indicated that, overall, species growing in disturbed ground showed a significant germination response to alternating temperature with a large amplitude (5/25°C), but not to alternating temperature with a small amplitude (10/20°C). Species living in alpine/subalpine meadow, forest margin /scrub, marshland and dry sunny slope habitats did not have a significant germination response to alternating temperature, regardless of the amplitude.

**Table 1:** Germination responses of species that differ in habitat, seed mass, life cycle type, altitudinal distribution and functional group.

Factor	Group	n	df	*x* ^*2*^	*p*
Habitat	Alpine/subalpine meadow	137	2	3.140	0.208
	Disturbed ground	91	2	11.029	0.004
	Forest margin/scrub	99	2	2.439	0.295
	Marshland	52	2	2.920	0.232
	Dry sunny slope	66	2	7.015	0.030
Seed mass (mg)	≤1	259	2	11.144	0.004
	1-2	82	2	6.939	0.031
	≥2	104	2	3.103	0.212
Life cycle	Annual/biennial	121	2	6.763	0.034
	Perennial	324	2	7.992	0.018
Altitudinal distribution (m)	≥2000	177	2	3.644	0.162
	<and ≥2000	268	2	10.755	0.005
Functional group	Graminoids	80	2	1.517	0.468
	Forbs	365	2	18.667	0.000

### Effect of seed mass on germination response to temperature fluctuation

A positive germination response to temperature fluctuation occurred in 39.00%, 40.24% and 25.00% of the species with a seed mass of < 1, 1-2 and > 2mg, respectively (Figure 2b). Our results (Table 1 Figure 3) indicated that, overall, species with an average seed mass < 1mg showed a significant positive germination response to temperature fluctuation, regardless of the temperature amplitude. Species with an average seed mass of 1-2mg had a positive germination response to alternating temperature with a large amplitude, but not to the alternating temperature with a small amplitude. Species with a seed mass >2mg did not have a significant germination response to alternating temperatures, regardless of amplitude.

### Effect of life cycle type on germination response to temperature fluctuation

A higher proportion of annuals/biennials responded positively to temperature fluctuation than perennials (Figure 2c). Overall, the annuals/biennials had a significant positive response to alternating temperature with a large amplitude but not to alternating temperature with a small amplitude (Table 1 Figure 3). Perennials had a significant positive response to alternating temperature, regardless of the amplitude (Table 1 Figure 3).

### Effect of altitudinal distribution on germination response to temperature fluctuation

Compared to15°C, 5/25°C increased the mean germination percentage of species that occur only at the high elevations (>2000m) from 39.41% to 43.43%, and 10/20 °C increased it from 39.41% to 42.27%. For species occurring at both high (>2000m) and low (<2000m) elevations, 5/25°C increased mean germination percentage from 39.34% to 45.65%, and 10/20°C increased it from 39.34% to 44.40% (Table 1 Figure 3d). The results (Table 1 Figure 3) indicated that, overall, species distributed only at high elevations did not have a significant germination response to the temperature fluctuation, regardless of the temperature amplitude. However, species occurring at both high and low elevations had a significant positive germination response to alternating temperature regimes with both a large amplitude and a small amplitude (Table 1 Figure 3).

### Effect of functional group on germination response to temperature fluctuation

A higher proportion of forbs (38.63%) than of graminoids (23.75%) responded positively to temperature fluctuation. Overall, temperature fluctuation did not have a significant effect on germination of graminoids (Table 1 Figure 3). However, it significantly increased the average germination of forbs (Table 1 Figure 3). Compared to constant temperature (15°C), alternating temperature with a large amplitude (5/25°C) increased the mean germination percentage of forbs from 35.67% to 42.51%, and alternating temperature with a small amplitude (10/20 °C) increased it from 35.67% to 40.94%.

## Discussion

Temperature fluctuation improved germination at the community level, and the germination response to temperature fluctuation was affected by seed mass, life cycle type, habitat, altitudinal distribution and functional group (Figure 3). Temperature fluctuation improved germination at the community level, and the germination response to temperature fluctuation was affected by seed mass, life cycle type, habitat, altitudinal distribution and functional group (Figure 3). However, not all the species have the same germination response to temperature fluctuation. Only the germination of species of group II responded positively to temperature fluctuation, species of group I had a negative germination response to temperature fluctuation and species of groups III and IV had no significant response to temperature fluctuation. This difference in germination response of different groups of species to temperature fluctuation indicated differentiation of the regeneration niche, which can help explain plant distribution and also the coexistence of species in the studied grassland. Species whose germination is enhanced by temperature fluctuation (e.g. species of group II) are likely to occur in gaps, whereas those whose germination is depressed by temperature (e.g. species of group I) are likely to occur under vegetation; as a result, these two types of species can coexist in a grassland with gaps.

### Effect of habitat on germination response to temperature fluctuation

A large proportion of the species living in disturbed ground, forest margin /scrub and marshland, and a smaller proportion of those living in alpine/subalpine meadow and dry sunny slope had a positive response to temperature fluctuation. These results are consistent with those of previous studies. Thompson and Grime [19] found that stimulation of germination of native herbaceous plants in the Sheffield area of England by alternating temperatures was strongly habitat-dependent. Germination in light of more than 40% of the wetland species was promoted by alternating temperature. Alternating-temperature promotion of germination in darkness is widespread in species of disturbed ground and to a lesser extent so are those of grasslands [6,19]. Species living in marshlands need to detect the water level in order to avoid germinating in deep water. Since shallow water and bare mud experience a much larger temperature fluctuation than deep water, a positive germination response to temperature fluctuation is a good mechanism to limit germination to shallow water or bare mud, where conditions are suitable for seedling establishment.

The undisturbed grasslands of the Tibet Plateau are dominated by species of *Kobresia* *, Carex, Poa, Elymus* and other perennial graminoids that may reproduce vegetatively, and it is very difficult for seedlings to become established in such closed turf [6,26]. Consequently seedling establishment is mostly limited to gaps. Thus, gap-detection is critical for recruitment of alpine/subalpine species. Also, many fugitive plants grow in disturbed ground, and they usually have poor competitive ability. Thus, it is unlikely that these species can survive the competition with species that live in undisturbed grassland; as a result, fugitive species can grow only in disturbed environments. Thus, a positive response to temperature fluctuation is a very good mechanism to ensure that these species germinate only in disturbed environments (where there usually are many gaps).

Compared with species in other habitats, those growing on dry sunny slopes were less likely to respond positively to temperature fluctuation and more likely to have a negative response to it. The Friedman Test also indicated that, overall, species on dry sunny slopes did not have a significant germination response to alternating temperature, regardless of the amplitude. Soil moisture is the main factor limiting seedling establishment of species occurring on dry sunny slopes. After the beginning of the growing season, seedling establishment in this habitat would be promoted by rapid germination as soon as the soil is moist, regardless of the daily temperature fluctuation. Additionally, in rainy weather soil moisture usually is suitable for seed germination, but the daily temperature fluctuation usually is small. Thus, not surprisingly, germination of many species on dry sunny slopes was depressed by temperature fluctuation.

A large proportion of species in forest margin /scrub can respond positively to temperature fluctuation. However, the Friedman Test did not show any overall significant promotion of germination for this group, due to the fact that many species living in this habitat are shade tolerant and have a negative response to temperature fluctuation.

### Effect of seed mass on germination response to temperature fluctuation

Germination of small-seeded species was more likely to be promoted by fluctuating temperatures than that of large-seeded species (Figure 3b). These effects are very similar to those of seed mass on germination response to light [27,28]. Seeds of small-seeded species are more sensitive to these germination cues than those of large seeded species. One explanation for the relationship between seed mass and germination response to temperature fluctuation is that seed size is positively related to seedling size [29–31], and small seedlings (from small seeds) are less likely to survive in competition with established plants than large seedlings (from large seeds). Thus, it is important for small-seeded species to germinate in competition-free sites (gaps) for successful seedling establishment. Germination promoted by fluctuating temperatures is a mechanism for gap-detection, thus germination of small-seeded species is more dependent on temperature fluctuation than that of large-seeded species. Additionally, small seeds are more likely to become buried [6,32,33], but seedlings of small seeds are much less likely to emerge from deep soil than those from large seeds [3]. Thus, depth sensing is much more important for small seeds than for large seeds. As a result, germination of small seeds is more sensitive to temperature fluctuations that occur at or near the soil surface. On the other hand, large seedlings can emerge from deep in the soil and can tolerate competition from neighbor plants; thus, large seeds do not necessarily respond to fluctuating temperature.

### Effect of life cycle type on germination response to temperature fluctuation

Taking all annuals/biennials into account, the alternating temperature regime with a large amplitude (20°C) promoted germination, but the one with a small amplitude (10°C) did not. However, overall, germination of the perennials was promoted by alternating temperatures, regardless of the amplitude. Annuals are much more dependent on seeds for regeneration each year than perennials. Thus, compared to perennials seeds of annuals and biennials might be expected to have a more selective germination requirement, which ensures that the environment is suitable for seedling establishment [18,34]. Compared to alternating temperatures with a small amplitude, those with a large amplitude can more accurately indicate the presence of the gaps and shallow soil depth. Thus, a positive response only to alternating temperature with large amplitude helps to ensure that germination will occur at places that are suitable for seedling establishment. Seed germination of the annuals/biennials was promoted only by alternating temperature with a large amplitude. The requirement for large temperature fluctuation to trigger germination contributes to the formation of the seed bank [6]. Persistent seed banks can buffer the dependence of annuals and biennials on seeds produced in a particular year.

### Effect of altitudinal distribution on germination response to temperature fluctuation

Lack of a response to temperature fluctuation of the species distributed only at high elevations may be an adaptive mechanism of plants to the high altitude environment. Average daily temperature fluctuation throughout the year on the Tibet Plateau ranges from about 12 to 20°C, depending on month and year, and it is much higher than that at lower elevation at a similar latitude (Table S3). On the Tibet Plateau, even under the vegetation and at soil depths of 5cm, the daily temperature fluctuation can be as high as 10°C (Liu, unpublished data). Given that the growing season is very short at high elevations and that a high amplitude of temperature fluctuation occurs in all months of the year (Table S3), fluctuating temperature per se may not be a reliable indicator of suitable conditions for seedling establishment and growth. Rather, the maximum daily temperature could be a cue that the short summer growing season has begun and the trigger for germination. Further, germination of these seeds at constant 15°C suggests that the maximum temperature for germination may be relatively low.

### Effect of functional group on germination response to temperature fluctuation

Graminoids and forbs are the primary functional groups of natural grasslands on the eastern Tibet Plateau. Graminoids in these grasslands are mainly comprised of Poaceae, Cyperaceae and Juncaceae. Meadow is the climax community, and the undisturbed meadows are dominated by graminoids. Compared to temperature fluctuation in gaps, temperature fluctuation under the closed graminoid turf is small. Lack of a positive germination response to temperature fluctuation is an adaptive strategy in meadows on the eastern Tibet Plateau. In the heavily disturbed and degraded meadow, the composition of forbs increased. This is because such disturbed vegetation usually has a lot of gaps. Additionally, positive germination response to temperature fluctuation gives forbs a mechanism to detect gaps, and absence of competition in gaps is very conducive to seedling establishment. Thus, forbs can play a role in heavily disturbed and degraded meadows.

## Conclusion

Our study was performed at the community level, and it gave us a broad view of how temperature fluctuation affects seed germination at the community level. For about 36% of the species in the studied grassland, the regeneration ability by seeds can be increased by temperature fluctuation. Overall, species growing in alpine/subalpine meadow, disturbed ground and marshland showed a significant germination response to temperature fluctuation, but species living in forest margin /scrub and dry sunny slope habitats did not. Species distributed only at high elevations (>2000m) did not show a significant germination response to temperature fluctuation, whereas those occurring at both high and low elevations had a significant positive response. Germination of annuals/biennials was significantly promoted by 5/25°C, but not by 10/20°C, whereas germination of perennials was significantly promoted by both 5/25°C and 10/20°C. Small-seeded species were more likely than large-seeded species to respond positively to fluctuating temperatures. Germination of forbs responded positively to temperature fluctuation, but germination of graminoids did not.

Previous observation indicated that the average diurnal temperature fluctuation will get smaller and smaller with global warming, because the rise of the minimum temperature has occurred at a rate three times that of the maximum temperature [35,36]. Additionally, our results indicated that the effect of temperature fluctuation on seed germination is species specific. Thus, we predict that the continuous decrease of diurnal temperature fluctuation (caused by global warming) may have some effect on community structure. Species whose germination is stimulated by temperature fluctuation may decrease in community, because diurnal temperature fluctuation will get smaller and smaller, and thus the germination requirements of these species cannot be fulfilled. However, this prediction requires additional studies, especially field studies. Stimulation of germination of forbs by temperature fluctuation gives us a reasonable explanation of why there are more forbs in degraded grassland (where there are many gaps with large diurnal temperature fluctuation near the soil surface) than in undisturbed grassland.

## Supporting Information

Table S1 Habitats, life cycle and altitudinal distribution of the 445species.
(DOC)

Table S2 Germination percentage, seed mass and functional group for species in the various germination response groups (I–IV). G_5/25_, germination percentage at 5/25°C; G_10/20_, germination percentage at 10/20 °C; G_15_, germination percentage at 15 °C; G, graminoids; F, forbs.
(DOC)

Table S3 Daily temperature fluctuation on the eastern Tibet Plateau (Maqu) and three other places with the similar latitude but different altitudes.
(DOC)
