# Supplementary material for: Effect of Diurnal Fluctuating versus Constant Temperatures on Germination of 445 Species from the Eastern Tibet Plateau
Source: PLoS One. 2013 Jul 24;8(7):e69364. doi: 10.1371/journal.pone.0069364 (PMC3722265; doi:10.1371/journal.pone.0069364)
Supplement: Table S1 — Habitats, life cycle and altitudinal distribution of the 445species. (DOC) [file pone.0069364.s001.doc]

**Table S1**. Habitats, life cycle and altitudinal distribution of the 445species.

| Species | Habitats | Life history | Altitude distribution | |
| --- | --- | --- | --- | --- |
| Achnatherum extremiorientale (Hara) Keng ex P. C. Kuo | dry sunny slops | perennial | ≥&<2000m | |
| Achnatherum inebrians (Hance) Keng | disturbed grounds | perennial | ≥&<2000m | |
| Achnatherum psilantherum Keng | alpine/subalpine meadows | perennial | ≥2000m | |
| Achnatherum sibiricum (L.) Keng ex Tzvel. | dry sunny slops | perennial | ≥&<2000m | |
| Achnatherum splendens (Trin.) Nevski | dry sunny slops | perennial | ≥&<2000m | |
| Aconitum gymnandrum Maxim. | disturbed grounds | annual/biennial | ≥&<2000m | |
| Aconitum sinomontanum Nakai | forest margins/scrubs | perennial | ≥&<2000m | |
| Acroglochin persicarioides (Poir.)Mog. | forest margins/scrubs | annual/biennial | ≥&<2000m | |
| Adenophora potaninii Korsh. | dry sunny slops | perennial | ≥&<2000m | |
| Adenophora stenanthina (Ledeb.) Kitag. | alpine/subalpine meadows | perennial | ≥&<2000m | |
| Agrimonia pilosa Ledeb | forest margins/scrubs | perennial | ≥&<2000m | |
| Agrostis gigantea Roth | alpine/subalpine meadows | perennial | ≥&<2000m | |
| Agrostis hugoniana Rendle | alpine/subalpine meadows | perennial | ≥2000m | |
| Agrostis perlaxa Pilger | marshlands | perennial | ≥2000m | |
| Ajania salicifolia (Mattf.) Poljak. | disturbed grounds | perennial | ≥2000m | |
| Ajania tenuifolia (Jocq.) Tzvel. | disturbed grounds | perennial | ≥2000m | |
| Ajuga lupulina Maxim. | disturbed grounds | perennial | ≥&<2000m | |
| Aletris alpestris Diels | forest margins/scrubs | perennial | ≥&<2000m | |
| Allium cyaneum Regel | alpine/subalpine meadows | perennial | ≥2000m | |
| Allium rude J.M.Xu | alpine/subalpine meadows | perennial | ≥2000m | |
| Allium victorialis L. | forest margins/scrubs | perennial | ≥&<2000m | |
| Amethystea caerulea L. | dry sunny slops | annual/biennial | ≥&<2000m | |
| Anaphalis aureo-punctata Lingelsh et Borza | forest margins/scrubs | perennial | ≥&<2000m | |
| Anaphalis flavescens Hand.-Mazz. | alpine/subalpine meadows | perennial | ≥2000m | |
| Anaphalis hancockii Maxim. | alpine/subalpine meadows | perennial | ≥2000m | |
| Anaphalis lactea Maxim. | alpine/subalpine meadows | perennial | ≥2000m | |
| Anaphalis latialata Ling et Y. L. Chen var. viridis (Hand.-Mazz.) Ling et Y.L.Chen | dry sunny slops | perennial | ≥2000m | |
| Anaphalis margaritacea (L.) Benth. et Hook. f. | disturbed grounds | perennial | ≥&<2000m | |
| Androsace erecta Maxim. | alpine/subalpine meadows | annual/biennial | ≥2000m | |
| Androsace gmelinii (Gaertn.) Roem. et Schuit. | marshlands | annual/biennial | ≥2000m | |
| Androsace mariae Kanitz. | dry sunny slops | perennial | ≥&<2000m | |
| Anemone rivularis Buch.-Ham. | alpine/subalpine meadows | perennial | ≥&<2000m | |
| Angelica nitida Wolff | alpine/subalpine meadows | perennial | ≥2000m | |
| Anisodus tanguticus (Maxinowicz) Pascher | disturbed grounds | perennial | ≥2000m | |
| Anthriscus sylvestris (L.) Hoffm. | disturbed grounds | perennial | ≥&<2000m | |
| Aquilegia ecalcarata Maxim. | forest margins/scrubs | perennial | ≥&<2000m | |
| Aquilegia oxysepala Trautv. et Mey. var. kansuensis Bruhl | disturbed grounds | perennial | ≥&<2000m | |
| Arabis pendula L. | forest margins/scrubs | annual/biennial | ≥&<2000m | |
| Arctium lappa L. | disturbed grounds | annual/biennial | ≥&<2000m | |
| Arenaria kansuensis Maxim. | alpine/subalpine meadows | perennial | ≥2000m | |
| Arenaria serpyllifolia L. | alpine/subalpine meadows | perennial | ≥&<2000m | |
| Arisaema erubescens (Wall.) Schott | forest margins/scrubs | perennial | ≥&<2000m | |
| Aristida triseta Keng | dry sunny slops | perennial | ≥2000m | |
| Artemisia argyi Lévl. et Vant. | disturbed grounds | perennial | ≥&<2000m | |
| Artemisia desertorum Spreng. var. tongolensis Pamp. | alpine/subalpine meadows | perennial | ≥2000m | |
| Artemisia dubia Wall. ex Bess. | disturbed grounds | perennial | ≥&<2000m | |
| Artemisia hedinii Ostenf. et Pauls. | disturbed grounds | annual/biennial | ≥2000m | |
| Artemisia mongolica (Fisch. ex Bess.) Nakai | disturbed grounds | perennial | ≥&<2000m | |
| Artemisia roxburghiana Bess. | disturbed grounds | perennial | ≥&<2000m | |
| Artemisia sacrorum Ledeb. | dry sunny slops | perennial | ≥&<2000m | |
| Artemisia scoparia Waldst. et Kit. | disturbed grounds | perennial | ≥&<2000m | |
| Artemisia sieversiana Ehrhart ex Willd. | disturbed grounds | annual/biennial | ≥&<2000m | |
| Asparagus longiflorus Franch. | forest margins/scrubs | perennial | ≥2000m | |
| Asperugo procumbens L. | disturbed grounds | annual/biennial | ≥2000m | |
| Aster albescens (DC.) Hand.-Mazz. var. limprichtii (Diels) Hand.-Mazz. | forest margins/scrubs | perennial | ≥2000m | |
| Aster diplostephioides (DC.) C.B.Clark | alpine/subalpine meadows | perennial | ≥2000m | |
| Aster farreri W. W. Sm. et J. F. Jeffr. | alpine/subalpine meadows | perennial | ≥2000m | |
| Aster poliothamnus Diels | dry sunny slops | perennial | ≥&<2000m | |
| Aster yunnanensis var. labrangensis (Hand.-Mazz.) Ling | alpine/subalpine meadows | perennial | ≥2000m | |
| Astragalus adsurgens Pall. | dry sunny slops | perennial | ≥&<2000m | |
| Astragalus bhotanensis Baker | forest margins/scrubs | perennial | ≥&<2000m | |
| Astragalus floridus Benth. ex Bunge | forest margins/scrubs | perennial | ≥2000m | |
| Astragalus melilotoides Pall. | dry sunny slops | perennial | ≥&<2000m | |
| Astragalus polycladus Bur. et Franch. | alpine/subalpine meadows | perennial | ≥2000m | |
| Astragalus przewalskii Bunge ex Maxim. | alpine/subalpine meadows | perennial | ≥2000m | |
| Astragalus skythropos Bunge | alpine/subalpine meadows | perennial | ≥2000m | |
| Astragalus tongolensis Ulbr. var. glaber Pet.-Stib. | forest margins/scrubs | perennial | ≥2000m | |
| Axyris amaranthoides L. | disturbed grounds | annual/biennial | ≥&<2000m | |
| Batrachium bungei (Steud.) L.Liou | marshlands | perennial | ≥&<2000m | |
| Beckmannia syzigachne (Steud.) Fern. | marshlands | annual/biennial | ≥&<2000m | |
| Bidens bipinnata L. | dry sunny slops | annual/biennial | ≥&<2000m | |
| Bidens tripartita L. | marshlands | annual/biennial | ≥&<2000m | |
| Brachypodium sylvaticum (Huds.) Beauv. | alpine/subalpine meadows | perennial | ≥&<2000m | |
| Bromus japonicus Thunb. ex Murr. | disturbed grounds | annual/biennial | ≥&<2000m | |
| Bromus magnus Keng | alpine/subalpine meadows | perennial | ≥2000m | |
| Bromus tectorum L. | dry sunny slops | annual/biennial | ≥&<2000m | |
| Bupleurum boissieuanum H.Wolff | forest margins/scrubs | perennial | ≥&<2000m | |
| Bupleurum commelynoideum H. de Boiss. | alpine/subalpine meadows | perennial | ≥2000m | |
| Bupleurum smithii H.Wolff | forest margins/scrubs | perennial | ≥&<2000m | |
| Calamagrostis pseudophragmites (Hall. F.) Koel. | disturbed grounds | perennial | ≥&<2000m | |
| Caltha palustris L. | marshlands | perennial | ≥&<2000m | |
| Caltha scaposa Hook. | marshlands | perennial | ≥2000m | |
| Capsella bursa-pastoris (L.) Medic. | disturbed grounds | annual/biennial | ≥&<2000m | |
| Cardamine impartiens L. var. dasycarpa (M.Bieb.) T.Y.Cheo et R.C.Fang | forest margins/scrubs | perennial | ≥&<2000m | |
| Carex chlorostachys Stev. | alpine/subalpine meadows | perennial | ≥&<2000m | |
| Carex coriophora Fisch. | marshlands | perennial | ≥&<2000m | |
| Carex enervis C.A.Mey. | alpine/subalpine meadows | perennial | ≥2000m | |
| Carex kansuensis Irelmes | alpine/subalpine meadows | perennial | ≥2000m | |
| Carex lehmanii Drejer | forest margins/scrubs | perennial | ≥2000m | |
| Carex scabrirostris Kukenth. | marshlands | perennial | ≥2000m | |
| Carpesium lipskyi Winkl. | forest margins/scrubs | perennial | ≥2000m | |
| Carum buriaticum Turcz.f. | alpine/subalpine meadows | perennial | ≥&<2000m | |
| Cerastium fontanum Baumg. subsp. triviale (Link) Jalas | disturbed grounds | perennial | ≥&<2000m | |
| Ceratoides arborescens (Losina-Losinskaja) Czerepanov | dry sunny slops | perennial | ≥&<2000m | |
| Chamaesium thalictrifolium Wolff | forest margins/scrubs | perennial | ≥2000m | |
| Chelidonium majus L. | disturbed grounds | perennial | ≥&<2000m | |
| Chenopodium album L. | disturbed grounds | annual/biennial | ≥&<2000m | |
| Chenopodium aristatum L. | disturbed grounds | annual/biennial | ≥&<2000m | |
| Chenopodium foetidum Schrad. | disturbed grounds | annual/biennial | ≥&<2000m | |
| Chenopodium glaucum L. | disturbed grounds | annual/biennial | ≥&<2000m | |
| Chenopodium prostratum Bunge | disturbed grounds | annual/biennial | ≥&<2000m | |
| Cimicifuga foetida L. | forest margins/scrubs | perennial | ≥&<2000m | |
| Circaeaster agrestis Maxim. | forest margins/scrubs | annual/biennial | ≥2000m | |
| Cirsium leo Nakai et Kitag. | disturbed grounds | perennial | ≥&<2000m | |
| Clematis brevicaudata DC. | forest margins/scrubs | perennial | ≥&<2000m | |
| Clematis tangutica (Maxim.) Korsh. | disturbed grounds | perennial | ≥&<2000m | |
| Cnidium monnieri (L.) Cuss. | disturbed grounds | perennial | ≥&<2000m | |
| Codonopsis canescens Nannf. | alpine/subalpine meadows | perennial | ≥2000m | |
| Codonopsis pilosula (Franch.) Nannf. | forest margins/scrubs | perennial | ≥&<2000m | |
| Coluria longifolia Maxim. | alpine/subalpine meadows | perennial | ≥2000m | |
| Comastoma pedunculatum (Royle ex D.Don) Holub | alpine/subalpine meadows | annual/biennial | ≥2000m | |
| Comastoma pulmonarium (Turcz.) Toyokuni | alpine/subalpine meadows | annual/biennial | ≥2000m | |
| Corispermum tibeticum Iljin | disturbed grounds | annual/biennial | ≥2000m | |
| Corydalis adunca Maxim. | dry sunny slops | perennial | ≥&<2000m | |
| Corydalis pseudoimpatiens Feddle | forest margins/scrubs | annual/biennial | ≥2000m | |
| Cremanthodium discoideum Maxim. | marshlands | perennial | ≥2000m | |
| Cucubalus baccifer L. | disturbed grounds | perennial | ≥&<2000m | |
| Cuscuta europaea L. | forest margins/scrubs | annual/biennial | ≥&<2000m | |
| Cuscuta japonica Choisy | disturbed grounds | annual/biennial | ≥&<2000m | |
| Cyananthus hookeri C.B.Clarke | alpine/subalpine meadows | annual/biennial | ≥2000m | |
| Cynanchum inamoenum (Maxim.) Loes. | forest margins/scrubs | perennial | ≥&<2000m | |
| Cynodon dactylon (L.) Pers. | disturbed grounds | perennial | ≥&<2000m | |
| Cynoglossum amabile Stapf et Drumm. | dry sunny slops | perennial | ≥2000m | |
| Delphinium albocoeruleum Maxim. | alpine/subalpine meadows | perennial | ≥&<2000m | |
| Delphinium kamaonense Hunth var. glabrescens (W.T.Wang) W.T.Wang | alpine/subalpine meadows | annual/biennial | ≥2000m | |
| Delphinium pylzowii Maxim. var. trigynum W.T.Wang | forest margins/scrubs | perennial | ≥2000m | |
| Delphinium siwanense Franch. var. leptopogon (Hand.-Mazz.) W.T.Wang | forest margins/scrubs | perennial | ≥&<2000m | |
| Deschampsia caespitosa (L.) Beauv. | marshlands | perennial | ≥&<2000m | |
| Descurainia sophia (L.) Webb ex Prantl | disturbed grounds | annual/biennial | ≥&<2000m | |
| Deyeuxia flavens Keng | alpine/subalpine meadows | perennial | ≥2000m | |
| Dianthus superbus L. | alpine/subalpine meadows | perennial | ≥&<2000m | |
| Dicranostigma lactucoides Hook. f. et Thoms. | dry sunny slops | perennial | ≥2000m | |
| Dicranostigma leptopodum (Maxim.) Fedde | dry sunny slops | perennial | ≥&<2000m | |
| Digitaria ciliaris (Retz.) Koel. | disturbed grounds | annual/biennial | ≥&<2000m | |
| Dipsacus japonicus Miq. | disturbed grounds | perennial | ≥&<2000m | |
| Draba eriopoda Turcz. | forest margins/scrubs | annual/biennial | ≥2000m | |
| Draba nemorosa L. | forest margins/scrubs | annual/biennial | ≥&<2000m | |
| Dracocephalum heterophyllum Benth | dry sunny slops | perennial | ≥&<2000m | |
| Dracocephalum tanguticum Maxim. | disturbed grounds | perennial | ≥2000m | |
| Duchesnea indica (Andr.) Focke | alpine/subalpine meadows | perennial | ≥&<2000m | |
| Echinochloa crusgali (L.) Beauv. var. mitis (Pursh) Peterm. | disturbed grounds | annual/biennial | ≥&<2000m | |
| Elsholtzia densa Benth. | disturbed grounds | annual/biennial | ≥&<2000m | |
| Elsholtzia densa Benth. var. calycocarpa (Diels)C.Y.Wu et S.C.Huang | disturbed grounds | annual/biennial | ≥&<2000m | |
| Elsholtzia fruticosa (D. Don) Rehd. | disturbed grounds | perennial | ≥&<2000m | |
| Elymus barystachyus A.Love. | alpine/subalpine meadows | perennial | ≥&<2000m | |
| Elymus cylindricus (Franch.) Honda | alpine/subalpine meadows | perennial | ≥&<2000m | |
| Elymus dahuricus Turcz. ex Griseb. | alpine/subalpine meadows | perennial | ≥&<2000m | |
| Elymus excelsus Turez. | alpine/subalpine meadows | perennial | ≥&<2000m | |
| Elymus sp | alpine/subalpine meadows | perennial | ≥&<2000m | |
| Elymus tangutorum (Neuski) Hand.-Mazz. | alpine/subalpine meadows | perennial | ≥&<2000m | |
| Epilobium angustifolium (L.) Scop. | forest margins/scrubs | perennial | ≥&<2000m | |
| Epilobium palustre L. | marshlands | perennial | ≥&<2000m | |
| Eragrostis nigra Nees ex Steud. | dry sunny slops | perennial | ≥&<2000m | |
| Erigeron acer L. | alpine/subalpine meadows | annual/biennial | ≥&<2000m | |
| Eruca sativa Mill. | disturbed grounds | annual/biennial | ≥&<2000m | |
| Euphorbia helioscopia L. | disturbed grounds | annual/biennial | ≥&<2000m | |
| Euphrasia regelii Wettst. | alpine/subalpine meadows | annual/biennial | ≥&<2000m | |
| Fallopia aubertii (L. Henry) Holub | forest margins/scrubs | perennial | ≥&<2000m | |
| Fallopia convolvula (L.) A. Love | alpine/subalpine meadows | annual/biennial | ≥&<2000m | |
| Festuca nitidula Stapf | alpine/subalpine meadows | perennial | ≥2000m | |
| Festuca ovina L. | alpine/subalpine meadows | perennial | ≥&<2000m | |
| Festuca rubra L. | alpine/subalpine meadows | perennial | ≥&<2000m | |
| Festuca sinensis Keng ex S.L.Lu | alpine/subalpine meadows | perennial | ≥2000m | |
| Fragaria orientalis Lozinsk. | forest margins/scrubs | perennial | ≥&<2000m | |
| Fritillaria unibracteata Hsiao et K. C. Hsia | alpine/subalpine meadows | perennial | ≥2000m | |
| Galeopsis bifida Boenn. | disturbed grounds | annual/biennial | ≥&<2000m | |
| Galium aparine L. var. echinospermum (Wallr.) Cuf. | disturbed grounds | annual/biennial | ≥&<2000m | |
| Galium verum L. | forest margins/scrubs | perennial | ≥&<2000m | |
| Gentiana abaensis T. N. Ho | alpine/subalpine meadows | annual/biennial | ≥2000m | |
| Gentiana choanantha Marq. | marshlands | annual/biennial | ≥2000m | |
| Gentiana crassuloides Bureau et Franch. | alpine/subalpine meadows | annual/biennial | ≥2000m | |
| Gentiana leucomelaena Maxim. | marshlands | annual/biennial | ≥&<2000m | |
| Gentiana pseudo-aquatica Kusnez. | marshlands | annual/biennial | ≥&<2000m | |
| Gentiana spathulifolia Maxim. ex Kusnez. | forest margins/scrubs | annual/biennial | ≥2000m | |
| Gentiana squarrosa Ledeb. | alpine/subalpine meadows | annual/biennial | ≥&<2000m | |
| Gentiana stipitata Edgew. | marshlands | perennial | ≥2000m | |
| Gentiana straminea Maxim. | alpine/subalpine meadows | perennial | ≥2000m | |
| Gentiana striata Maxim. | alpine/subalpine meadows | annual/biennial | ≥2000m | |
| Gentianopsis contorta (Royle) Maxim. | forest margins/scrubs | annual/biennial | ≥&<2000m | |
| Gentianopsis paludosa (Hook. f.) Ma | marshlands | annual/biennial | ≥&<2000m | |
| Gentianopsis paludosa var. Ovatodeltoidea (Burk.) Ma ex T.N.Ho | alpine/subalpine meadows | annual/biennial | ≥&<2000m | |
| Geum aleppicum Jacq. | alpine/subalpine meadows | perennial | ≥&<2000m | |
| Halenia elliptica D.Don | forest margins/scrubs | annual/biennial | ≥&<2000m | |
| Hedysarum multijugum Maxim. | dry sunny slops | perennial | ≥&<2000m | |
| Hedysarum polybotrys Hand.-Mazz. | forest margins/scrubs | perennial | ≥&<2000m | |
| Hedysarum tanguticum B.Fedtsch. | alpine/subalpine meadows | perennial | ≥2000m | |
| Helictotrichon tibeticum (Rasheu.) Holub | alpine/subalpine meadows | perennial | ≥2000m | |
| Heracleum millefolium Diels | disturbed grounds | perennial | ≥2000m | |
| Heracleum moellendorffii Hance | forest margins/scrubs | perennial | ≥&<2000m | |
| Heteropappus altaicus (Willd.) Novopokr. | dry sunny slops | perennial | ≥&<2000m | |
| Heteropappus crenatifolius (Hand.-Mazz.) Griers. | disturbed grounds | annual/biennial | ≥2000m | |
| Heteropappus gouldii (C. E. C. Fisch.) Griers. | alpine/subalpine meadows | annual/biennial | ≥2000m | |
| Hierochloe laxa R. Br. ex Hook. f. | forest margins/scrubs | perennial | ≥2000m | |
| Hippuris vulgaris L. | marshlands | perennial | ≥&<2000m | |
| Hylotelephium angustum (Maxim.) H. Ohba | marshlands | perennial | ≥&<2000m | |
| Hyoscyamus niger L. | disturbed grounds | annual/biennial | ≥&<2000m | |
| Hypericum ascyron L. | forest margins/scrubs | perennial | ≥&<2000m | |
| Hypericum przewalskii Maxim. | marshlands | perennial | ≥2000m | |
| Impatiens noli-tangere L. | forest margins/scrubs | annual/biennial | ≥&<2000m | |
| Incarvillea compacta Maxim. | dry sunny slops | perennial | ≥2000m | |
| Incarvillea sinensis Lam. | dry sunny slops | annual/biennial | ≥&<2000m | |
| Incarvillea sinensis Lam. var. przewalskii (Batalin) C.Y.Wu et W.C.Yi | dry sunny slops | annual/biennial | ≥&<2000m | |
| Indigofera silvestrii Pamp. | dry sunny slops | perennial | ≥&<2000m | |
| Iris lactea Pall. | alpine/subalpine meadows | perennial | ≥&<2000m | |
| Juncus allioides Franch. | marshlands | perennial | ≥&<2000m | |
| Juncus bufonius L. | marshlands | annual/biennial | ≥&<2000m | |
| Juncus himalensis Klotzsch | marshlands | perennial | ≥2000m | |
| Juncus potaninii Buchen. | marshlands | perennial | ≥2000m | |
| Juncus prismatocarpus R. Brown | marshlands | perennial | ≥&<2000m | |
| Juncus thomsonii Buchen. | marshlands | perennial | ≥2000m | |
| Kalimeris mongolica (Franch.) Kitam. | forest margins/scrubs | perennial | ≥&<2000m | |
| Kobresia kansuensis Kükenth. | marshlands | perennial | ≥2000m | |
| Kobresia myosuroides (Villas) Fiori | marshlands | perennial | ≥2000m | |
| Kobresia pygmaea C.B.Clarke | alpine/subalpine meadows | perennial | ≥2000m | |
| Kobresia tibetica Maximowicz | alpine/subalpine meadows | perennial | ≥2000m | |
| Kochia scoparia (L.) Schrad. | disturbed grounds | annual/biennial | ≥&<2000m | |
| Koeleria litvinowii Dom. | alpine/subalpine meadows | perennial | ≥2000m | |
| Lagotis brachystachya Maxim. | disturbed grounds | perennial | ≥2000m | |
| Lamium amplexicaule L. | disturbed grounds | annual/biennial | ≥&<2000m | |
| Lancea tibetica Hook.f. et Thoms. | disturbed grounds | perennial | ≥2000m | |
| Lathyrus pratensis L. | forest margins/scrubs | perennial | ≥&<2000m | |
| Leibnitzia nepalensis (Kunze) Kitamura | alpine/subalpine meadows | perennial | ≥&<2000m | |
| Leontopodium haplophylloides Hand.-Mazz. | alpine/subalpine meadows | perennial | ≥2000m | |
| Leontopodium leontopodioides (Willd.) Beauv. | dry sunny slops | perennial | ≥&<2000m | |
| Leontopodium souliei Beauv. | alpine/subalpine meadows | perennial | ≥2000m | |
| Leonurus japonicus Houtt. | disturbed grounds | annual/biennial | ≥&<2000m | |
| Lepidium apetalum Willdenow | disturbed grounds | annual/biennial | ≥&<2000m | |
| Lepidium cuneiforme C. Y. Wu | dry sunny slops | annual/biennial | ≥&<2000m | |
| Lepyrodiclis holosteoides (C. A. Meyer) Fenzl. ex Fisher et C. A. Meyer | disturbed grounds | annual/biennial | ≥&<2000m | |
| Leymus secalinus (Georgi) Tzvel. | alpine/subalpine meadows | perennial | ≥2000m | |
| Ligularia fischeri (Ledeb.) Turcz. | forest margins/scrubs | perennial | ≥&<2000m | |
| Ligularia mocrodouta Ling | forest margins/scrubs | perennial | ≥2000m | |
| Ligularia przewalskii (Maxim.) Diels | forest margins/scrubs | perennial | ≥&<2000m | |
| Ligularia sagitta (Maxim.) Mattf. | alpine/subalpine meadows | perennial | ≥&<2000m | |
| Ligularia veitchiana (Hemsl.) Greenm. | forest margins/scrubs | perennial | ≥&<2000m | |
| Ligularia virgaurea (Maxim.) Mattf. | marshlands | perennial | ≥2000m | |
| Ligusticum thomsonii C.B.Clarke | forest margins/scrubs | perennial | ≥2000m | |
| Lilium pumilum DC. | dry sunny slops | perennial | ≥&<2000m | |
| Linum amurense Alef. | dry sunny slops | perennial | ≥&<2000m | |
| Linum perenne L. | dry sunny slops | perennial | ≥&<2000m | |
| Lloydia oxycarpa Franch. | alpine/subalpine meadows | perennial | ≥2000m | |
| Lomatogonium carinthiacum (Wulf.) Reichb. | alpine/subalpine meadows | annual/biennial | ≥&<2000m | |
| Lomatogonium macranthum (Diels et Gilg) Fern. | marshlands | annual/biennial | ≥2000m | |
| Malcolmia africana (L.) R.Br. | disturbed grounds | annual/biennial | ≥&<2000m | |
| Malva verticillata L. var. chinensis (Miller) S.Y.Hu | disturbed grounds | annual/biennial | ≥&<2000m | |
| Meconopsis horridula Hook. f. et Thoms. | disturbed grounds | annual/biennial | ≥2000m | |
| Meconopsis integrifolia (Maxim.) Franch. | alpine/subalpine meadows | annual/biennial | ≥2000m | |
| Meconopsis punicea Maxim. | alpine/subalpine meadows | perennial | ≥2000m | |
| Meconopsis quintuplinervia Regel | alpine/subalpine meadows | perennial | ≥2000m | |
| Medicago archiducis-nicolai Sirj. | alpine/subalpine meadows | perennial | ≥2000m | |
| Medicago lupulina L. | dry sunny slops | perennial | ≥&<2000m | |
| Medicago ruthenica (L.) Trautv. | forest margins/scrubs | perennial | ≥&<2000m | |
| Medicago varia Martyn. | disturbed grounds | perennial | ≥&<2000m | |
| Megacarpaea delavayi Franch. | alpine/subalpine meadows | perennial | ≥2000m | |
| Melandrium apricum (Turcz. ex Fisch. et. Mey.) Rohrb. | dry sunny slops | annual/biennial | ≥&<2000m | |
| Melica onoei French et Sav. | forest margins/scrubs | perennial | ≥&<2000m | |
| Melilotus officinalis (L.) Desr. | disturbed grounds | annual/biennial | ≥&<2000m | |
| Morina nepalensis D. Don var. alba (Hand.-Mazz.) Y.C.Tang | alpine/subalpine meadows | perennial | ≥2000m | |
| Nardostachys chinensis Bat. | marshlands | perennial | ≥2000m | |
| Nepeta prattii Lévl. | alpine/subalpine meadows | perennial | ≥&<2000m | |
| Notopterygium forbesii Boiss. | forest margins/scrubs | perennial | ≥&<2000m | |
| Notopterygium incisum Ting ex H. T. Chang | forest margins/scrubs | perennial | ≥&<2000m | |
| Orostachys fimbriatus (Turcz.) Berger | dry sunny slops | annual/biennial | ≥&<2000m | |
| Oxalis corniculata L. | dry sunny slops | perennial | ≥&<2000m | |
| Oxygraphis glacialis (Fisch.) Bunge | alpine/subalpine meadows | perennial | ≥2000m | |
| Oxytropis falcata Bunge | dry sunny slops | perennial | ≥2000m | |
| Oxytropis kansuensis Bunge | alpine/subalpine meadows | perennial | ≥2000m | |
| Oxytropis ochrocephala Bunge | alpine/subalpine meadows | perennial | ≥&<2000m | |
| Oxytropis xinglongshanica C.W.Chang | dry sunny slops | perennial | ≥&<2000m | |
| Paraixeris denticulata (Houtt.) Nakai | forest margins/scrubs | annual/biennial | ≥2000m | |
| Paraquilegia microphylla (Royle) Drumm. et Hutch. | marshlands | perennial | ≥2000m | |
| Parasenecio deltophyllus (Maxim.) Y. L. Chen | forest margins/scrubs | perennial | ≥2000m |  |
| Parasenecio roborowskii (Maxim.) Y. L. Chen | forest margins/scrubs | perennial | ≥&<2000m | |
| Parinia heterophylla Bunge | dry sunny slops | perennial | ≥&<2000m | |
| Parnassia oreophila Hance | forest margins/scrubs | perennial | ≥&<2000m | |
| Parnassia trinervis Drude | alpine/subalpine meadows | perennial | ≥2000m | |
| Pedicularis alaschanica Maxim. | dry sunny slops | perennial | ≥2000m | |
| Pedicularis cheilanthifolia Schrenk | marshlands | perennial | ≥2000m | |
| Pedicularis chinensis Maxim. | alpine/subalpine meadows | annual/biennial | ≥&<2000m | |
| Pedicularis kansuensis Maxim. | alpine/subalpine meadows | annual/biennial | ≥&<2000m | |
| Pedicularis lachnoglossa Hk. F. | alpine/subalpine meadows | perennial | ≥2000m | |
| Pedicularis lasiophrys Maxim. | alpine/subalpine meadows | perennial | ≥2000m | |
| Pedicularis lasiophrys Maxim. var. sinica Maxim. | alpine/subalpine meadows | perennial | ≥2000m | |
| Pedicularis longiflora Rudolph var. tubiformis （Klotz.） Tsoong | marshlands | annual/biennial | ≥2000m | |
| Pedicularis polyodonta Li | alpine/subalpine meadows | annual/biennial | ≥2000m | |
| Pedicularis rudis Maxim. | forest margins/scrubs | perennial | ≥2000m | |
| Pedicularis semitorta Maxim. | alpine/subalpine meadows | annual/biennial | ≥2000m | |
| Pedicularis spicata Pall. | forest margins/scrubs | annual/biennial | ≥&<2000m | |
| Pedicularis striata Pall. subsp. arachnoidea (Franch.) Tsoong | dry sunny slops | perennial | ≥&<2000m | |
| Pedicularis ternata Maxim. | alpine/subalpine meadows | perennial | ≥2000m | |
| Pedicularis tristis L. | alpine/subalpine meadows | perennial | ≥2000m | |
| Pedicularis cristatella Pennell et Li | forest margins/scrubs | annual/biennial | ≥&<2000m | |
| Peganum multisectum (Maxim.) Bobr. | dry sunny slops | perennial | ≥&<2000m | |
| Pennisetum centrasiaticum Tzvel. | dry sunny slops | perennial | ≥&<2000m | |
| Pertya discolor Rehd. | forest margins/scrubs | perennial | ≥&<2000m | |
| Phlomis umbrosa Turcz. | forest margins/scrubs | perennial | ≥&<2000m | |
| Phragmites australis (Cav.) Trin. ex Steud. | marshlands | perennial | ≥&<2000m | |
| Phtheirospermum japonicum (Thunb.) Kanitz | dry sunny slops | annual/biennial | ≥&<2000m | |
| Phytolacca acinosa Roxb. | disturbed grounds | perennial | ≥&<2000m | |
| Picris hieracioides L. ssp. Japonica Krylv. | disturbed grounds | annual/biennial | ≥&<2000m | |
| Plantago asiatica L. | disturbed grounds | annual/biennial | ≥&<2000m | |
| Plantago depressa Willd. | disturbed grounds | annual/biennial | ≥&<2000m | |
| Pleurospermum cristatum H.de Boiss | forest margins/scrubs | annual/biennial | ≥&<2000m | |
| Plumbagella micrantha (Ledeb.) Spach | disturbed grounds | annual/biennial | ≥2000m | |
| Poa annua L. | marshlands | perennial | ≥&<2000m | |
| Poa attenuata Trin. | dry sunny slops | perennial | ≥2000m | |
| Poa attenuata Trin. var. vivipara Rendle | alpine/subalpine meadows | perennial | ≥2000m | |
| Poa crymophila Keng ex C.Ling | alpine/subalpine meadows | perennial | ≥2000m | |
| Poa declinata Keng ex L.Liou | alpine/subalpine meadows | perennial | ≥2000m | |
| Poa paucifolia Keng | dry sunny slops | perennial | ≥&<2000m | |
| Poa pratensis L. | marshlands | perennial | ≥&<2000m | |
| Poa tunicata Keng | alpine/subalpine meadows | annual/biennial | ≥2000m | |
| Polemonium coeruleum L. var. chinense Brand | forest margins/scrubs | perennial | ≥2000m | |
| Polygonatum verticillatum (L.) All. | forest margins/scrubs | perennial | ≥2000m | |
| Polygonum fertile (Maxim.) A.J.Li | alpine/subalpine meadows | annual/biennial | ≥2000m | |
| Polygonum hydropiper L. | marshlands | annual/biennial | ≥&<2000m | |
| Polygonum macrophyllum D. Don | alpine/subalpine meadows | perennial | ≥2000m | |
| Polygonum orientale L | disturbed grounds | annual/biennial | ≥&<2000m | |
| Polygonum sibiricum L. | disturbed grounds | perennial | ≥&<2000m | |
| Polygonum sparsipilosum A.J.L | marshlands | annual/biennial | ≥2000m | |
| Polypogon fugax Nees ex Steud. | marshlands | annual/biennial | ≥&<2000m | |
| Pomatosace filicula Maxim. | disturbed grounds | annual/biennial | ≥2000m | |
| Potentilla bifurca L. | disturbed grounds | perennial | ≥&<2000m | |
| Potentilla chinensis Ser. | dry sunny slops | perennial | ≥&<2000m | |
| Potentilla conferta Bunge | alpine/subalpine meadows | perennial | ≥&<2000m | |
| Potentilla longifolia Willd. ex Schlecht. | forest margins/scrubs | perennial | ≥&<2000m | |
| Potentilla multifolia L. | alpine/subalpine meadows | perennial | ≥&<2000m | |
| Potentilla potaninii Wolf | alpine/subalpine meadows | perennial | ≥&<2000m | |
| Potentilla supina L. | alpine/subalpine meadows | annual/biennial | ≥&<2000m | |
| Potentilla tanacetifolia Willd. ex Schlecht. | dry sunny slops | perennial | ≥&<2000m | |
| Prenanthes macrophylla Franch. | forest margins/scrubs | perennial | ≥&<2000m | |
| Prenanthes tatarinowii Maxim. | forest margins/scrubs | perennial | ≥&<2000m | |
| Primula gemmifera Batal. | marshlands | perennial | ≥2000m | |
| Primula nutans Georgi | marshlands | perennial | ≥&<2000m | |
| Primula orbicularis Hemsl. | alpine/subalpine meadows | perennial | ≥2000m | |
| Primula tangutica Duthie | marshlands | perennial | ≥2000m | |
| Ptilagrostis concinna (Hook. f.) Roshev. | alpine/subalpine meadows | perennial | ≥2000m | |
| Ptilagrostis dichotoma Keng ex Tzvel. | alpine/subalpine meadows | perennial | ≥2000m | |
| Pycreus sanguinolentus (Vahl) Nees | marshlands | annual/biennial | ≥&<2000m | |
| Pyrethrum tatsienense (Bur. et Franch.) Ling ex Shih | alpine/subalpine meadows | perennial | ≥2000m | |
| Ranunculus tanguticus (Maxim.) Ovcz. | marshlands | perennial | ≥2000m | |
| Rhodiola dumulosa (Franch.) S.H.Fu | dry sunny slops | perennial | ≥&<2000m | |
| Rhodiola eurycarpa (Fröd.) S.H.Fu | forest margins/scrubs | perennial | ≥2000m | |
| Rhodiola kirilowii (Regel) Maxim. | alpine/subalpine meadows | perennial | ≥2000m | |
| Rodgersia aesculifolia Batal. | forest margins/scrubs | perennial | ≥&<2000m | |
| Roegneria breviglumis Keng | alpine/subalpine meadows | perennial | ≥2000m | |
| Roegneria dura (Keng) Keng | alpine/subalpine meadows | perennial | ≥2000m | |
| Roegneria kokonorica Keng | dry sunny slops | perennial | ≥2000m | |
| Roegneria stricta Keng | dry sunny slops | perennial | ≥&<2000m | |
| Roegneria varia Keng et S.L.Cheng | forest margins/scrubs | perennial | ≥2000m | |
| Rorippa palustris (L.) Bess. | marshlands | annual/biennial | ≥&<2000m | |
| Rumex crispus L. | disturbed grounds | perennial | ≥&<2000m | |
| Rumex nepalensis Spreng. | alpine/subalpine meadows | perennial | ≥&<2000m | |
| Rumex patientia L. | disturbed grounds | perennial | ≥&<2000m | |
| Rumex pseudonatronatus (Borb.) Borb. ex Murb. | forest margins/scrubs | perennial | ≥&<2000m | |
| Salvia przewalskii Maxim. | disturbed grounds | perennial | ≥&<2000m | |
| Salvia roborowskii Maxim. | disturbed grounds | annual/biennial | ≥2000m | |
| Sambucus chinensis Lindl. | forest margins/scrubs | perennial | ≥&<2000m | |
| Sanguisorba officinalis L. | alpine/subalpine meadows | perennial | ≥&<2000m | |
| Saussurea globosa Chen | alpine/subalpine meadows | perennial | ≥2000m | |
| Saussurea hieracioides Hook.f. | alpine/subalpine meadows | perennial | ≥2000m | |
| Saussurea iodostegia Hance | alpine/subalpine meadows | perennial | ≥&<2000m | |
| Saussurea japonica (Thunb.) DC. | dry sunny slops | annual/biennial | ≥&<2000m | |
| Saussurea macrota Franch. | forest margins/scrubs | perennial | ≥2000m | |
| Saussurea parviflora (Poir.) DC. | forest margins/scrubs | perennial | ≥&<2000m | |
| Saussurea stella Maxim. | marshlands | annual/biennial | ≥2000m | |
| Saussurea sylvatica Maxim. | forest margins/scrubs | perennial | ≥2000m | |
| Saussurea variiloba Ling | forest margins/scrubs | perennial | ≥&<2000m | |
| Saxifraga egregia Engl. | alpine/subalpine meadows | perennial | ≥2000m | |
| Saxifraga montana H. Smith | alpine/subalpine meadows | perennial | ≥2000m | |
| Schizonepeta multifida (L.) Briq. | forest margins/scrubs | perennial | ≥&<2000m | |
| Scirpus distigmaticus (Kükenth.) Tang et Wang | alpine/subalpine meadows | perennial | ≥2000m | |
| Scorzonera austriaca Willd. | dry sunny slops | perennial | ≥&<2000m | |
| Scrofella chinensis Maxim. | alpine/subalpine meadows | perennial | ≥2000m | |
| Scrophularia incisa Weinm. | disturbed grounds | perennial | ≥&<2000m | |
| Scutellaria baicalensis Georgi | dry sunny slops | perennial | ≥&<2000m | |
| Sedum aizoon L. | forest margins/scrubs | perennial | ≥&<2000m | |
| Senecio argunensis Turcz. | forest margins/scrubs | perennial | ≥&<2000m | |
| Senecio densiserratus Chang | forest margins/scrubs | perennial | ≥2000m | |
| Senecio dubitabilis C.Jeffrey et Y.L.Chen | disturbed grounds | annual/biennial | ≥2000m | |
| Seseli squarrulosum Shan et Sheh | dry sunny slops | perennial | ≥&<2000m | |
| Setaria glauca (L.) Beauv. | disturbed grounds | annual/biennial | ≥&<2000m | |
| Setaria viridis (L.) Beauv. | disturbed grounds | annual/biennial | ≥&<2000m | |
| Sibbaldia procumbens L. var. aphanopetala (Hand.-Mazz.) Yü et Li | alpine/subalpine meadows | perennial | ≥2000m | |
| Siegesbeckia pubescens Makino | disturbed grounds | annual/biennial | ≥&<2000m | |
| Silene conoidea L. | disturbed grounds | annual/biennial | ≥&<2000m | |
| Silene fortunei Vis | disturbed grounds | perennial | ≥&<2000m | |
| Silene gracilicaulis C. L. Tang | dry sunny slops | perennial | ≥2000m | |
| Silene pterosperma Maxim. | dry sunny slops | perennial | ≥2000m | |
| Silene repens Patr. | disturbed grounds | perennial | ≥&<2000m | |
| Sinochasea trigyna Keng | alpine/subalpine meadows | perennial | ≥2000m | |
| Sinopodophyllum hexandrum (Royle) Ying | forest margins/scrubs | perennial | ≥2000m | |
| Sinosenecio euosmus (Hand.-Mazz.) B. Nord. | forest margins/scrubs | perennial | ≥2000m | |
| Sisymbrium heteromallum C.A.Mey. | forest margins/scrubs | annual/biennial | ≥&<2000m | |
| Solanum alatum Moench | disturbed grounds | perennial | ≥&<2000m | |
| Sorosers erysimoides (Hand-Mazz) Shih. | alpine/subalpine meadows | perennial | ≥2000m | |
| Souliea vaginata (Maxim.) Franch. | forest margins/scrubs | perennial | ≥2000m | |
| Sphallerocarpus gracilis (Trevir) K.-Pol. | disturbed grounds | perennial | ≥&<2000m | |
| Stachys sieboldi Miq. | marshlands | perennial | ≥&<2000m | |
| Stellaria dianthifolia Williams | forest margins/scrubs | perennial | ≥2000m | |
| Stellaria graminea L. | forest margins/scrubs | perennial | ≥&<2000m | |
| Stellaria neglecta Weihe ex Fingerh. | disturbed grounds | annual/biennial | ≥&<2000m | |
| Stellaria parviumbellata Y. Z. Zhao | dry sunny slops | perennial | ≥&<2000m | |
| Stellera chamaejasme L. | dry sunny slops | perennial | ≥2000m | |
| Stephanachne nigrescens Keng | alpine/subalpine meadows | perennial | ≥2000m | |
| Stipa bungeana Trin. | dry sunny slops | perennial | ≥&<2000m | |
| Stipa przewalskyi Roshev. | alpine/subalpine meadows | perennial | ≥&<2000m | |
| Swertia bifolia Batal. | alpine/subalpine meadows | perennial | ≥2000m | |
| Swertia bimaculata (Sieb.et Zucc.)Hook.f.et Thoms. ex Clarke | marshlands | annual/biennial | ≥&<2000m | |
| Swertia diluta (Turcz.)Benth. et Hook. f. | forest margins/scrubs | annual/biennial | ≥&<2000m | |
| Swertia erythrosticta Maxim. | marshlands | perennial | ≥&<2000m | |
| Swertia franchetiana H. Smith | alpine/subalpine meadows | annual/biennial | ≥2000m | |
| Swertia tetraptera Maxim. | alpine/subalpine meadows | annual/biennial | ≥2000m | |
| Taraxacum calanthodium Dahlst. | alpine/subalpine meadows | perennial | ≥2000m | |
| Taraxacum maurocarpum Dahlst. | alpine/subalpine meadows | perennial | ≥2000m | |
| Taraxacum mongolicum Hand.-Mazz. | disturbed grounds | perennial | ≥&<2000m | |
| Thalictrum alpinum L. var. elatum Ulbr. | alpine/subalpine meadows | perennial | ≥2000m | |
| Thalictrum macrorhynchum Franch. | forest margins/scrubs | perennial | ≥&<2000m | |
| Thalictrum minus L. | forest margins/scrubs | perennial | ≥&<2000m | |
| Thalictrum minus L. var. hypoleucum (Sieb.et Zucc.) Miq. | forest margins/scrubs | perennial | ≥&<2000m | |
| Thalictrum przewalskii Maxim. | forest margins/scrubs | perennial | ≥&<2000m | |
| Thalictrum rutifolium Hook.f. et Thoms. | forest margins/scrubs | perennial | ≥2000m | |
| Thalictrum uncatum Maxim. | forest margins/scrubs | perennial | ≥&<2000m | |
| Thermopsis lanceolata R. Br. | dry sunny slops | perennial | ≥&<2000m | |
| Thesium refractum C.A.Mey. | dry sunny slops | perennial | ≥&<2000m | |
| Thymus mongolicus Ronn. | dry sunny slops | perennial | ≥&<2000m | |
| Tibetia himalaica (Baker) Tsui | alpine/subalpine meadows | perennial | ≥2000m | |
| Tongoloa elata Wolff | alpine/subalpine meadows | perennial | ≥2000m | |
| Tragus berteronianus Schult. | dry sunny slops | annual/biennial | ≥&<2000m | |
| Triglochin palustre L. | marshlands | perennial | ≥&<2000m | |
| Trisetum clarkei (Hook. f.) R.R.Stewart | forest margins/scrubs | perennial | ≥&<2000m | |
| Vaccaria segetalis (Neck.) Garcke | disturbed grounds | annual/biennial | ≥&<2000m | |
| Valeriana officinalis L. | alpine/subalpine meadows | perennial | ≥&<2000m | |
| Verbena officinalis L. | disturbed grounds | perennial | ≥&<2000m | |
| Veronica anagallisaquatica L. | marshlands | perennial | ≥&<2000m | |
| Veronica ciliata Fisch. | alpine/subalpine meadows | perennial | ≥2000m | |
| Veronica eriogyne H.Winkl. | alpine/subalpine meadows | perennial | ≥2000m | |
| Veronica rockii H.L.Li | alpine/subalpine meadows | perennial | ≥&<2000m | |
| Veronica szechuanica Batal. | alpine/subalpine meadows | annual/biennial | ≥&<2000m | |
| Vicia angustifolia L. ex Reichard | disturbed grounds | annual/biennial | ≥&<2000m | |
| Vicia cracca L. | forest margins/scrubs | perennial | ≥&<2000m | |
| Vicia multicaulis Ledeb. | forest margins/scrubs | perennial | ≥&<2000m | |
| Vicia unijuga A.Br. | forest margins/scrubs | perennial | ≥&<2000m | |
| Xanthopappus subacaulis C. Winkl. | dry sunny slops | perennial | ≥2000m | |
| Zygophyllum mucronatum Maxim. | dry sunny slops | perennial | ≥&<2000m | |
