# Supplementary material for: Effect of Diurnal Fluctuating versus Constant Temperatures on Germination of 445 Species from the Eastern Tibet Plateau
Source: PLoS One. 2013 Jul 24;8(7):e69364. doi: 10.1371/journal.pone.0069364 (PMC3722265; doi:10.1371/journal.pone.0069364)
Supplement: Table S2 — Germination percentage, seed mass and functional group for species in the various germination response groups (I–IV). G5/25, germination percentage at 5/25°C; G10/20, germination percentage at 10/20 °C; G15, germination percentage at 15 °C; G, graminoids; F, forbs. (DOC) [file pone.0069364.s002.doc]

**Table S2**. Germination percentage, seed mass and functional group for species in the various germination response groups (I-IV) . G_5/25_, germination percentage at 5/25°C; G_10/20_, germination percentage at 10/20 °C; G_15_, germination percentage at 15 °C; G, graminoids; F, forbs.

| Species | G_5/25_ (%) | G_10/20_ (%) | G_15_ (%) | groups | Seed mass (mg) | Functional group |
| --- | --- | --- | --- | --- | --- | --- |
| Achnatherum extremiorientale (Hara) Keng ex P. C. Kuo | 58.33 | 80.00 | 92.67 | I | 2.6223 | G |
| Achnatherum inebrians (Hance) Keng | 96.00 | 98.67 | 98.67 | IV | 1.3700 | G |
| Achnatherum psilantherum Keng | 96.67 | 99.33 | 90.00 | IV | 1.3613 | G |
| Achnatherum sibiricum (L.) Keng ex Tzvel. | 80.67 | 91.33 | 67.33 | II | 3.1803 | G |
| Achnatherum splendens (Trin.) Nevski | 69.33 | 84.00 | 82.67 | IV | 0.2865 | G |
| Aconitum gymnandrum Maxim. | 54.67 | 60.00 | 28.67 | II | 2.4660 | F |
| Aconitum sinomontanum Nakai | 0.67 | 1.33 | 2.90 | III | 2.1093 | F |
| Acroglochin persicarioides (Poir.)Mog. | 28.67 | 34.67 | 20.67 | II | 0.5990 | F |
| Adenophora potaninii Korsh. | 88.67 | 92.67 | 78.00 | IV | 0.1223 | F |
| Adenophora stenanthina (Ledeb.) Kitag. | 94.67 | 65.33 | 70.83 | II | 0.0940 | F |
| Agrimonia pilosa Ledeb | 76.67 | 78.67 | 6.67 | II | 8.6967 | F |
| Agrostis gigantea Roth | 90.67 | 98.00 | 62.00 | II | 0.1130 | G |
| Agrostis hugoniana Rendle | 91.33 | 59.33 | 64.67 | II | 0.1467 | G |
| Agrostis perlaxa Pilger | 62.00 | 76.67 | 74.00 | I | 0.1170 | G |
| Ajania salicifolia (Mattf.) Poljak. | 50.00 | 90.67 | 58.67 | II | 0.2217 | F |
| Ajania tenuifolia (Jocq.) Tzvel. | 67.33 | 55.33 | 62.00 | IV | 0.0807 | F |
| Ajuga lupulina Maxim. | 0.00 | 3.33 | 0.00 | III | 1.5703 | F |
| Aletris alpestris Diels | 18.00 | 0.00 | 0.00 | III | 0.0173 | F |
| Allium cyaneum Regel | 93.33 | 98.00 | 42.00 | II | 0.4913 | F |
| Allium rude J.M.Xu | 73.33 | 74.00 | 79.33 | IV | 0.5127 | F |
| Allium victorialis L. | 0.67 | 3.33 | 34.67 | I | 3.0203 | F |
| Amethystea caerulea L. | 34.67 | 2.67 | 2.67 | II | 0.5500 | F |
| Anaphalis aureo-punctata Lingelsh et Borza | 80.67 | 81.33 | 92.00 | IV | 0.0447 | F |
| Anaphalis flavescens Hand.-Mazz. | 66.67 | 92.67 | 92.00 | I | 0.1477 | F |
| Anaphalis hancockii Maxim. | 88.67 | 93.33 | 82.67 | IV | 0.1327 | F |
| Anaphalis lactea Maxim. | 97.33 | 92.00 | 99.33 | IV | 0.0943 | F |
| Anaphalis latialata Ling et Y. L. Chen var. viridis (Hand.-Mazz.) Ling et Y.L.Chen | 88.33 | 98.33 | 94.67 | IV | 0.0947 | F |
| Anaphalis margaritacea (L.) Benth. et Hook. f. | 95.33 | 96.00 | 96.00 | IV | 0.0787 | F |
| Androsace erecta Maxim. | 88.00 | 92.00 | 100.00 | IV | 0.0423 | F |
| Androsace gmelinii (Gaertn.) Roem. et Schuit. | 75.00 | 26.67 | 1.33 | II | 0.2360 | F |
| Androsace mariae Kanitz. | 19.33 | 31.33 | 94.00 | I | 0.4857 | F |
| Anemone rivularis Buch.-Ham. | 14.00 | 18.00 | 1.33 | III | 5.4660 | F |
| Angelica nitida Wolff | 1.33 | 0.00 | 1.33 | III | 1.8780 | F |
| Anisodus tanguticus (Maxinowicz) Pascher | 87.33 | 65.33 | 24.00 | II | 6.1540 | F |
| Anthriscus sylvestris (L.) Hoffm. | 2.67 | 0.00 | 13.33 | III | 3.3393 | F |
| Aquilegia ecalcarata Maxim. | 16.67 | 0.67 | 0.00 | III | 1.0947 | F |
| Aquilegia oxysepala Trautv. et Mey. var. kansuensis Bruhl | 5.33 | 0.00 | 0.00 | III | 1.1467 | F |
| Arabis pendula L. | 68.00 | 13.33 | 17.33 | II | 0.2890 | F |
| Arctium lappa L. | 91.33 | 42.67 | 73.33 | I | 11.5480 | F |
| Arenaria kansuensis Maxim. | 73.33 | 81.33 | 85.33 | I | 0.1257 | F |
| Arenaria serpyllifolia L. | 97.33 | 91.50 | 85.33 | IV | 0.0733 | F |
| Arisaema erubescens (Wall.) Schott | 82.67 | 91.33 | 66.67 | II | 11.8093 | F |
| Aristida triseta Keng | 28.00 | 54.00 | 91.33 | I | 0.2767 | G |
| Artemisia argyi Lévl. et Vant. | 56.67 | 54.00 | 96.67 | I | 0.1653 | F |
| Artemisia desertorum Spreng. var. tongolensis Pamp. | 68.00 | 65.67 | 92.50 | I | 0.2273 | F |
| Artemisia dubia Wall. ex Bess. | 92.67 | 97.33 | 94.00 | IV | 0.1073 | F |
| Artemisia hedinii Ostenf. et Pauls. | 99.33 | 99.33 | 98.00 | IV | 0.0800 | F |
| Artemisia mongolica (Fisch. ex Bess.) Nakai | 23.33 | 60.00 | 52.00 | II | 0.1167 | F |
| Artemisia roxburghiana Bess. | 93.33 | 92.67 | 54.67 | II | 0.1610 | F |
| Artemisia sacrorum Ledeb. | 6.00 | 2.00 | 0.00 | III | 0.1297 | F |
| Artemisia scoparia Waldst. et Kit. | 85.33 | 95.33 | 88.67 | IV | 0.0417 | F |
| Artemisia sieversiana Ehrhart ex Willd. | 44.67 | 82.67 | 94.67 | I | 0.1840 | F |
| Asparagus longiflorus Franch. | 2.67 | 19.33 | 0.67 | II | 29.5437 | F |
| Asperugo procumbens L. | 60.67 | 73.33 | 29.33 | II | 2.1500 | F |
| Aster albescens (DC.) Hand.-Mazz. var. limprichtii (Diels) Hand.-Mazz. | 76.00 | 82.67 | 68.00 | II | 0.3107 | F |
| Aster diplostephioides (DC.) C.B.Clark | 47.33 | 51.33 | 80.00 | I | 0.6627 | F |
| Aster farreri W. W. Sm. et J. F. Jeffr. | 93.33 | 94.00 | 14.67 | II | 0.5567 | F |
| Aster poliothamnus Diels | 68.00 | 93.33 | 48.89 | II | 0.3777 | F |
| Aster yunnanensis var. labrangensis (Hand.-Mazz.) Ling | 100.00 | 98.33 | 24.00 | II | 0.2977 | F |
| Astragalus adsurgens Pall. | 9.33 | 8.67 | 14.00 | III | 1.1487 | F |
| Astragalus bhotanensis Baker | 0.67 | 0.67 | 4.67 | III | 1.7287 | F |
| Astragalus floridus Benth. ex Bunge | 11.33 | 22.00 | 10.00 | III | 1.7703 | F |
| Astragalus melilotoides Pall. | 24.67 | 14.67 | 21.33 | III | 1.4403 | F |
| Astragalus polycladus Bur. et Franch. | 64.67 | 46.67 | 16.00 | II | 1.2370 | F |
| Astragalus przewalskii Bunge ex Maxim. | 8.00 | 3.00 | 8.67 | III | 2.2467 | F |
| Astragalus skythropos Bunge | 21.67 | 21.67 | 12.67 | II | 1.8087 | F |
| Astragalus tongolensis Ulbr. var. glaber Pet.-Stib. | 10.00 | 5.33 | 6.67 | III | 2.1297 | F |
| Axyris amaranthoides L. | 62.67 | 72.00 | 6.67 | II | 0.9370 | F |
| Batrachium bungei (Steud.) L.Liou | 51.33 | 52.67 | 0.00 | II | 0.4107 | F |
| Beckmannia syzigachne (Steud.) Fern. | 0.00 | 2.00 | 26.00 | I | 0.3663 | G |
| Bidens bipinnata L. | 65.33 | 89.33 | 88.67 | I | 4.1897 | F |
| Bidens tripartita L. | 9.33 | 3.33 | 58.00 | I | 3.8214 | F |
| Brachypodium sylvaticum (Huds.) Beauv. | 56.67 | 53.33 | 96.00 | I | 2.9167 | G |
| Bromus japonicus Thunb. ex Murr. | 99.33 | 98.67 | 92.00 | IV | 2.2130 | G |
| Bromus magnus Keng | 87.33 | 99.33 | 96.67 | IV | 2.0523 | G |
| Bromus tectorum L. | 98.00 | 100.00 | 98.67 | IV | 3.3413 | G |
| Bupleurum boissieuanum H.Wolff | 0.00 | 48.00 | 4.67 | II | 4.0520 | F |
| Bupleurum commelynoideum H. de Boiss. | 48.67 | 69.33 | 76.00 | I | 1.4307 | F |
| Bupleurum smithii H.Wolff | 96.67 | 94.00 | 90.00 | IV | 0.9017 | F |
| Calamagrostis pseudophragmites (Hall. F.) Koel. | 13.67 | 14.00 | 70.67 | I | 0.1940 | G |
| Caltha palustris L. | 0.00 | 0.00 | 0.00 | III | 0.3930 | F |
| Caltha scaposa Hook. | 0.00 | 0.00 | 2.00 | III | 0.0560 | F |
| Capsella bursa-pastoris (L.) Medic. | 96.67 | 58.67 | 10.00 | II | 0.0697 | F |
| Cardamine impartiens L. var. dasycarpa (M.Bieb.) T.Y.Cheo et R.C.Fang | 30.00 | 4.67 | 46.00 | I | 1.1933 | F |
| Carex chlorostachys Stev. | 70.67 | 0.00 | 0.00 | II | 0.3433 | G |
| Carex coriophora Fisch. | 2.00 | 0.00 | 1.33 | III | 0.5907 | G |
| Carex enervis C.A.Mey. | 0.00 | 0.00 | 0.67 | III | 0.5143 | G |
| Carex kansuensis Irelmes | 4.67 | 0.00 | 0.00 | III | 0.9247 | G |
| Carex lehmanii Drejer | 1.33 | 0.67 | 0.00 | III | 0.6823 | G |
| Carex scabrirostris Kukenth. | 0.00 | 0.67 | 0.00 | III | 1.9557 | G |
| Carpesium lipskyi Winkl. | 94.67 | 57.33 | 70.67 | II | 0.7267 | F |
| Carum buriaticum Turcz.f. | 16.67 | 42.00 | 78.00 | I | 0.8867 | F |
| Cerastium fontanum Baumg. subsp. triviale (Link) Jalas | 16.67 | 1.33 | 58.67 | I | 0.1787 | F |
| Ceratoides arborescens (Losina-Losinskaja) Czerepanov | 71.67 | 62.50 | 98.67 | I | 0.4193 | F |
| Chamaesium thalictrifolium Wolff | 0.00 | 0.00 | 0.00 | III | 0.9887 | F |
| Chelidonium majus L. | 84.67 | 34.00 | 35.56 | II | 1.2443 | F |
| Chenopodium album L. | 59.33 | 50.00 | 3.33 | II | 0.8230 | F |
| Chenopodium aristatum L. | 36.00 | 18.00 | 9.33 | II | 0.1113 | F |
| Chenopodium foetidum Schrad. | 87.33 | 84.67 | 34.67 | II | 0.1357 | F |
| Chenopodium glaucum L. | 17.33 | 14.00 | 92.67 | I | 0.2363 | F |
| Chenopodium prostratum Bunge | 26.67 | 25.33 | 4.00 | II | 0.4193 | F |
| Cimicifuga foetida L. | 0.67 | 0.00 | 0.00 | III | 0.0300 | F |
| Circaeaster agrestis Maxim. | 6.00 | 10.67 | 32.67 | III | 1.7310 | F |
| Cirsium leo Nakai et Kitag. | 36.00 | 6.67 | 28.00 | I | 4.6987 | F |
| Clematis brevicaudata DC. | 0.00 | 0.00 | 73.33 | I | 1.2550 | F |
| Clematis tangutica (Maxim.) Korsh. | 74.00 | 68.00 | 32.00 | II | 1.4997 | F |
| Cnidium monnieri (L.) Cuss. | 26.00 | 0.00 | 10.00 | II | 1.1287 | F |
| Codonopsis canescens Nannf. | 38.00 | 22.67 | 72.67 | I | 0.4057 | F |
| Codonopsis pilosula (Franch.) Nannf. | 47.33 | 68.67 | 20.67 | II | 0.2383 | F |
| Coluria longifolia Maxim. | 41.33 | 40.00 | 7.07 | II | 1.2803 | F |
| Comastoma pedunculatum (Royle ex D.Don) Holub | 0.67 | 0.67 | 0.00 | III | 0.1900 | F |
| Comastoma pulmonarium (Turcz.) Toyokuni | 45.33 | 23.33 | 2.67 | II | 0.2830 | F |
| Corispermum tibeticum Iljin | 29.33 | 48.67 | 44.00 | III | 1.2120 | F |
| Corydalis adunca Maxim. | 2.50 | 59.33 | 0.00 | II | 0.8500 | F |
| Corydalis pseudoimpatiens Feddle | 0.00 | 0.00 | 0.00 | III | 0.5420 | F |
| Cremanthodium discoideum Maxim. | 58.33 | 20.00 | 56.67 | I | 1.0976 | F |
| Cucubalus baccifer L. | 33.33 | 2.00 | 0.00 | II | 1.0927 | F |
| Cuscuta europaea L. | 2.00 | 0.67 | 0.67 | III | 0.3673 | F |
| Cuscuta japonica Choisy | 0.67 | 3.33 | 30.00 | I | 7.4683 | F |
| Cyananthus hookeri C.B.Clarke | 70.67 | 91.33 | 71.33 | II | 0.2657 | F |
| Cynanchum inamoenum (Maxim.) Loes. | 5.33 | 36.00 | 58.00 | I | 5.7693 | F |
| Cynodon dactylon (L.) Pers. | 4.00 | 0.00 | 0.00 | III | 0.2507 | G |
| Cynoglossum amabile Stapf et Drumm. | 1.67 | 26.67 | 98.00 | I | 5.9770 | F |
| Delphinium albocoeruleum Maxim. | 90.00 | 97.33 | 90.67 | IV | 0.8250 | F |
| Delphinium kamaonense Hunth var. glabrescens (W.T.Wang) W.T.Wang | 83.33 | 73.33 | 75.33 | IV | 0.5133 | F |
| Delphinium pylzowii Maxim. var. trigynum W.T. Wang | 74.00 | 72.00 | 22.00 | II | 0.4893 | F |
| Delphinium siwanense Franch. var. leptopogon (Hand.-Mazz.) W.T.Wang | 67.33 | 67.33 | 35.33 | II | 0.3683 | F |
| Deschampsia caespitosa (L.) Beauv. | 89.58 | 78.00 | 72.00 | II | 0.2420 | G |
| Descurainia sophia (L.) Webb ex Prantl | 97.33 | 99.33 | 25.33 | II | 0.1657 | F |
| Deyeuxia flavens Keng | 6.00 | 33.33 | 80.67 | I | 0.2650 | G |
| Dianthus superbus L. | 94.67 | 99.33 | 38.46 | II | 0.6607 | F |
| Dicranostigma lactucoides Hook. f. et Thoms. | 16.00 | 7.33 | 0.00 | III | 0.7147 | F |
| Dicranostigma leptopodum (Maxim.) Fedde | 48.00 | 5.33 | 9.52 | II | 0.6487 | F |
| Digitaria ciliaris (Retz.) Koel. | 24.67 | 52.00 | 14.67 | II | 0.4617 | G |
| Dipsacus japonicus Miq. | 0.00 | 0.00 | 0.00 | III | 6.4233 | F |
| Draba eriopoda Turcz. | 32.00 | 64.00 | 27.33 | II | 0.2353 | F |
| Draba nemorosa L. | 0.00 | 0.00 | 64.67 | I | 0.0203 | F |
| Dracocephalum heterophyllum Benth | 56.67 | 77.33 | 42.22 | II | 2.2440 | F |
| Dracocephalum tanguticum Maxim. | 60.00 | 46.67 | 21.33 | II | 1.8353 | F |
| Duchesnea indica (Andr.) Focke | 68.00 | 10.67 | 0.00 | II | 0.4923 | F |
| Echinochloa crusgali (L.) Beauv. var. mitis (Pursh) Peterm. | 82.22 | 36.67 | 87.62 | I | 1.5403 | G |
| Elsholtzia densa Benth. | 70.00 | 64.67 | 58.00 | II | 1.7647 | F |
| Elsholtzia densa Benth. var. calycocarpa (Diels)C.Y.Wu et S.C.Huang | 44.67 | 28.00 | 46.00 | I | 1.5450 | F |
| Elsholtzia fruticosa (D. Don) Rehd. | 88.00 | 88.67 | 42.67 | II | 0.0963 | F |
| Elymus barystachyus A.Love. | 83.33 | 95.00 | 51.33 | II | 6.1263 | G |
| Elymus cylindricus (Franch.) Honda | 77.33 | 91.33 | 97.33 | IV | 1.1533 | G |
| Elymus dahuricus Turcz. ex Griseb. | 82.67 | 79.33 | 36.67 | II | 3.7283 | G |
| Elymus excelsus Turez. | 100.00 | 100.00 | 87.33 | IV | 3.4603 | G |
| Elymus sp | 76.00 | 96.00 | 76.00 | IV | 3.7170 | G |
| Elymus tangutorum (Neuski) Hand.-Mazz. | 90.00 | 53.33 | 82.00 | I | 3.1620 | G |
| Epilobium angustifolium (L.) Scop. | 16.67 | 38.67 | 13.33 | II | 0.0587 | F |
| Epilobium palustre L. | 8.00 | 16.00 | 2.00 | III | 0.0997 | F |
| Eragrostis nigra Nees ex Steud. | 38.67 | 24.00 | 62.67 | I | 0.1123 | G |
| Erigeron acer L. | 86.67 | 78.67 | 94.67 | IV | 0.0887 | F |
| Eruca sativa Mill. | 97.33 | 97.33 | 92.67 | IV | 1.9177 | F |
| Euphorbia helioscopia L. | 3.33 | 55.33 | 39.33 | II | 0.9953 | F |
| Euphrasia regelii Wettst. | 0.00 | 0.00 | 0.67 | III | 0.0940 | F |
| Fallopia aubertii (L. Henry) Holub | 10.00 | 6.00 | 13.33 | III | 3.6760 | F |
| Fallopia convolvula (L.) A. Love | 0.67 | 0.00 | 5.56 | III | 2.9311 | F |
| Festuca nitidula Stapf | 52.67 | 68.67 | 88.67 | I | 0.5533 | G |
| Festuca ovina L. | 60.00 | 54.00 | 82.00 | I | 0.3857 | G |
| Festuca rubra L. | 51.33 | 42.00 | 76.67 | I | 0.6833 | G |
| Festuca sinensis Keng ex S.L.Lu | 91.33 | 86.67 | 98.00 | IV | 0.8723 | G |
| Fragaria orientalis Lozinsk. | 58.00 | 25.33 | 0.67 | II | 0.3760 | F |
| Fritillaria unibracteata Hsiao et K. C. Hsia | 0.00 | 0.00 | 0.00 | III | 0.7800 | F |
| Galeopsis bifida Boenn. | 0.67 | 0.67 | 3.33 | III | 3.2590 | F |
| Galium aparine L. var. echinospermum (Wallr.) Cuf. | 42.00 | 44.67 | 36.67 | II | 6.8643 | F |
| Galium verum L. | 94.00 | 68.67 | 31.33 | II | 0.7997 | F |
| Gentiana abaensis T. N. Ho | 0.00 | 2.67 | 6.00 | III | 0.0333 | F |
| Gentiana choanantha Marq. | 2.00 | 0.67 | 0.00 | III | 0.0740 | F |
| Gentiana crassuloides Bureau et Franch. | 8.00 | 3.33 | 0.00 | III | 0.0733 | F |
| Gentiana leucomelaena Maxim. | 2.67 | 22.00 | 1.33 | III | 0.0477 | F |
| Gentiana pseudo-aquatica Kusnez. | 0.67 | 56.00 | 64.67 | I | 0.0333 | F |
| Gentiana spathulifolia Maxim. ex Kusnez. | 50.67 | 8.00 | 0.00 | II | 0.0897 | F |
| Gentiana squarrosa Ledeb. | 88.00 | 50.00 | 6.00 | II | 0.0453 | F |
| Gentiana stipitata Edgew. | 51.33 | 56.00 | 64.00 | I | 0.1370 | F |
| Gentiana straminea Maxim. | 34.67 | 18.00 | 84.00 | I | 0.1697 | F |
| Gentiana striata Maxim. | 31.33 | 21.33 | 0.00 | II | 0.5823 | F |
| Gentianopsis contorta (Royle) Maxim. | 78.00 | 70.67 | 0.67 | II | 0.0770 | F |
| Gentianopsis paludosa (Hook. f.) Ma | 0.00 | 1.33 | 0.67 | III | 0.0913 | F |
| Gentianopsis paludosa var. Ovatodeltoidea (Burk.) Ma ex T.N.Ho | 1.33 | 0.00 | 0.67 | III | 0.0477 | F |
| Geum aleppicum Jacq. | 98.00 | 58.67 | 0.00 | II | 0.8807 | F |
| Halenia elliptica D.Don | 74.67 | 3.33 | 0.00 | II | 1.3483 | F |
| Hedysarum multijugum Maxim. | 86.00 | 86.00 | 96.67 | IV | 6.1970 | F |
| Hedysarum polybotrys Hand.-Mazz. | 23.33 | 23.33 | 60.00 | I | 3.7467 | F |
| Hedysarum tanguticum B.Fedtsch. | 4.67 | 13.33 | 20.00 | III | 2.9833 | F |
| Helictotrichon tibeticum (Rasheu.) Holub | 63.33 | 70.00 | 37.33 | II | 2.1340 | G |
| Heracleum millefolium Diels | 8.67 | 49.33 | 7.33 | II | 2.9453 | F |
| Heracleum moellendorffii Hance | 0.00 | 0.00 | 0.00 | III | 5.0297 | F |
| Heteropappus altaicus (Willd.) Novopokr. | 92.67 | 98.67 | 60.00 | II | 0.3103 | F |
| Heteropappus crenatifolius (Hand.-Mazz.) Griers. | 97.00 | 98.67 | 90.67 | IV | 0.3850 | F |
| Heteropappus gouldii (C. E. C. Fisch.) Griers. | 27.33 | 77.33 | 94.00 | I | 0.2680 | F |
| Hierochloe laxa R. Br. ex Hook. f. | 74.00 | 57.33 | 6.67 | II | 1.0137 | G |
| Hippuris vulgaris L. | 0.00 | 0.00 | 0.00 | III | 1.0967 | F |
| Hylotelephium angustum (Maxim.) H. Ohba | 13.33 | 0.00 | 0.00 | III | 0.0589 | F |
| Hyoscyamus niger L. | 98.00 | 18.00 | 0.00 | II | 0.5433 | F |
| Hypericum ascyron L. | 10.00 | 32.67 | 0.00 | II | 0.1270 | F |
| Hypericum przewalskii Maxim. | 22.67 | 40.18 | 1.33 | II | 0.1703 | F |
| Impatiens noli-tangere L. | 0.67 | 0.00 | 0.00 | III | 38.7740 | F |
| Incarvillea compacta Maxim. | 0.00 | 4.67 | 4.67 | III | 1.7547 | F |
| Incarvillea sinensis Lam. | 23.33 | 5.00 | 80.67 | I | 0.5263 | F |
| Incarvillea sinensis Lam. var. przewalskii (Batalin) C.Y.Wu et W.C.Yi | 25.33 | 52.00 | 48.00 | I | 0.4293 | F |
| Indigofera silvestrii Pamp. | 13.33 | 8.00 | 0.00 | III | 1.0727 | F |
| Iris lactea Pall. | 0.00 | 0.00 | 0.00 | III | 16.6047 | F |
| Juncus allioides Franch. | 0.00 | 0.00 | 12.67 | III | 0.0227 | G |
| Juncus bufonius L. | 0.00 | 1.33 | 0.00 | III | 0.0203 | G |
| Juncus himalensis Klotzsch | 96.67 | 21.33 | 16.00 | II | 0.0360 | G |
| Juncus potaninii Buchen. | 0.00 | 0.00 | 0.67 | III | 0.0243 | G |
| Juncus prismatocarpus R. Brown | 0.00 | 0.00 | 0.00 | III | 0.0160 | G |
| Juncus thomsonii Buchen. | 0.67 | 0.00 | 3.33 | III | 0.0473 | G |
| Kalimeris mongolica (Franch.) Kitam. | 62.00 | 79.33 | 88.89 | I | 0.6990 | F |
| Kobresia kansuensis Kükenth. | 82.67 | 38.67 | 24.00 | II | 1.1063 | G |
| Kobresia myosuroides (Villas) Fiori | 4.00 | 8.00 | 4.00 | III | 1.4343 | G |
| Kobresia pygmaea C.B.Clarke | 41.11 | 1.33 | 0.00 | II | 0.5990 | G |
| Kobresia tibetica Maximowicz | 23.33 | 4.67 | 3.33 | II | 1.5170 | G |
| Kochia scoparia (L.) Schrad. | 64.00 | 27.33 | 87.33 | I | 0.1660 | F |
| Koeleria litvinowii Dom. | 40.00 | 30.00 | 52.00 | I | 0.2673 | G |
| Lagotis brachystachya Maxim. | 9.33 | 9.33 | 57.33 | I | 11.6950 | F |
| Lamium amplexicaule L. | 2.00 | 22.00 | 2.67 | III | 0.6373 | F |
| Lancea tibetica Hook.f. et Thoms. | 30.00 | 0.67 | 0.00 | III | 0.0997 | F |
| Lathyrus pratensis L. | 6.67 | 10.00 | 5.33 | III | 11.8020 | F |
| Leibnitzia nepalensis (Kunze) Kitamura | 88.00 | 16.67 | 80.00 | I | 1.2920 | F |
| Leontopodium haplophylloides Hand.-Mazz. | 92.00 | 94.67 | 84.67 | IV | 0.0723 | F |
| Leontopodium leontopodioides (Willd.) Beauv. | 93.33 | 95.33 | 83.33 | IV | 0.0677 | F |
| Leontopodium souliei Beauv. | 86.00 | 89.33 | 92.67 | IV | 0.1157 | F |
| Leonurus japonicus Houtt. | 51.33 | 70.00 | 37.33 | II | 1.2630 | F |
| Lepidium apetalum Willdenow | 97.33 | 100.00 | 57.33 | II | 0.1850 | F |
| Lepidium cuneiforme C. Y. Wu | 99.33 | 100.67 | 100.00 | IV | 0.2063 | F |
| Lepyrodiclis holosteoides (C. A. Meyer) Fenzl. ex Fisher et C. A. Meyer | 12.67 | 21.33 | 2.67 | II | 1.8693 | F |
| Leymus secalinus (Georgi) Tzvel. | 94.67 | 98.00 | 76.67 | IV | 1.3093 | G |
| Ligularia fischeri (Ledeb.) Turcz. | 69.33 | 69.33 | 58.00 | II | 1.2323 | F |
| Ligularia mocrodouta Ling | 68.67 | 57.33 | 20.00 | II | 0.7723 | F |
| Ligularia przewalskii (Maxim.) Diels | 96.00 | 92.00 | 64.67 | II | 0.9643 | F |
| Ligularia sagitta (Maxim.) Mattf. | 86.00 | 88.67 | 82.67 | IV | 0.8067 | F |
| Ligularia veitchiana (Hemsl.) Greenm. | 63.33 | 90.00 | 43.33 | II | 1.0640 | F |
| Ligularia virgaurea (Maxim.) Mattf. | 80.00 | 79.33 | 58.67 | II | 1.5197 | F |
| Ligusticum thomsonii C.B.Clarke | 30.67 | 8.00 | 1.33 | II | 0.9243 | F |
| Lilium pumilum DC. | 30.00 | 78.67 | 90.67 | I | 2.5947 | F |
| Linum amurense Alef. | 62.67 | 80.00 | 82.00 | I | 1.2143 | F |
| Linum perenne L. | 93.33 | 87.33 | 92.67 | IV | 2.0687 | F |
| Lloydia oxycarpa Franch. | 23.33 | 14.67 | 20.00 | III | 0.5330 | F |
| Lomatogonium carinthiacum (Wulf.) Reichb. | 57.33 | 64.67 | 0.67 | II | 0.1273 | F |
| Lomatogonium macranthum (Diels et Gilg) Fern. | 67.33 | 48.00 | 2.00 | II | 0.2487 | F |
| Malcolmia africana (L.) R.Br. | 21.33 | 46.67 | 12.00 | II | 0.2993 | F |
| Malva verticillata L. var. chinensis (Miller) S.Y.Hu | 32.67 | 10.00 | 24.67 | I | 2.6170 | F |
| Meconopsis horridula Hook. f. et Thoms. | 22.67 | 65.33 | 58.00 | I | 0.1773 | F |
| Meconopsis integrifolia (Maxim.) Franch. | 0.00 | 0.00 | 0.00 | III | 0.5263 | F |
| Meconopsis punicea Maxim. | 0.00 | 0.67 | 0.00 | III | 0.5317 | F |
| Meconopsis quintuplinervia Regel | 0.00 | 0.00 | 0.00 | III | 0.8135 | F |
| Medicago archiducis-nicolai Sirj. | 16.67 | 19.33 | 18.00 | III | 2.1030 | F |
| Medicago lupulina L. | 4.00 | 6.00 | 6.67 | III | 2.1547 | F |
| Medicago ruthenica (L.) Trautv. | 10.67 | 16.67 | 6.00 | III | 2.5510 | F |
| Medicago varia Martyn. | 16.67 | 16.67 | 22.22 | III | 1.6580 | F |
| Megacarpaea delavayi Franch. | 6.67 | 1.33 | 2.67 | III | 6.8110 | F |
| Melandrium apricum (Turcz. ex Fisch. et. Mey.) Rohrb. | 100.00 | 93.33 | 99.33 | IV | 0.1900 | F |
| Melica onoei French et Sav. | 99.33 | 98.00 | 75.33 | IV | 0.9677 | G |
| Melilotus officinalis (L.) Desr. | 11.33 | 4.00 | 50.00 | I | 2.2010 | F |
| Morina nepalensis D. Don var. alba (Hand.-Mazz.) Y.C.Tang | 35.33 | 23.33 | 16.00 | II | 4.9333 | F |
| Nardostachys chinensis Bat. | 26.00 | 36.67 | 44.00 | I | 4.6023 | F |
| Nepeta prattii Lévl. | 9.33 | 4.67 | 38.67 | I | 1.2670 | F |
| Notopterygium forbesii Boiss. | 44.00 | 10.00 | 0.00 | II | 1.4347 | F |
| Notopterygium incisum Ting ex H. T. Chang | 0.00 | 0.00 | 0.00 | III | 2.5457 | F |
| Orostachys fimbriatus (Turcz.) Berger | 64.00 | 90.00 | 92.67 | I | 0.0347 | F |
| Oxalis corniculata L. | 78.67 | 94.00 | 52.00 | II | 0.2050 | F |
| Oxygraphis glacialis (Fisch.) Bunge | 80.00 | 65.00 | 6.00 | II | 2.4067 | F |
| Oxytropis falcata Bunge | 12.00 | 6.67 | 8.00 | III | 12.4093 | F |
| Oxytropis kansuensis Bunge | 4.00 | 4.67 | 8.00 | III | 0.9643 | F |
| Oxytropis ochrocephala Bunge | 9.33 | 11.33 | 1.33 | III | 1.5677 | F |
| Oxytropis xinglongshanica C.W.Chang | 4.67 | 9.33 | 0.67 | III | 4.9870 | F |
| Paraixeris denticulata (Houtt.) Nakai | 57.33 | 49.33 | 76.00 | I | 0.1133 | F |
| Paraquilegia microphylla (Royle) Drumm. et Hutch. | 30.00 | 29.33 | 0.00 | II | 0.2647 | F |
| Parasenecio deltophyllus (Maxim.) Y. L. Chen | 76.00 | 74.67 | 36.11 | II | 1.1943 | F |
| Parasenecio roborowskii (Maxim.) Y. L. Chen | 0.00 | 93.33 | 40.67 | II | 1.0057 | F |
| Parinia heterophylla Bunge | 75.33 | 60.67 | 11.33 | II | 1.2333 | F |
| Parnassia oreophila Hance | 0.67 | 0.00 | 1.33 | I | 0.0270 | F |
| Parnassia trinervis Drude | 85.33 | 68.00 | 46.00 | II | 0.0187 | F |
| Pedicularis alaschanica Maxim. | 60.67 | 12.00 | 0.00 | II | 0.9357 | F |
| Pedicularis cheilanthifolia Schrenk | 40.67 | 36.67 | 30.00 | II | 0.6513 | F |
| Pedicularis chinensis Maxim. | 10.67 | 0.00 | 2.00 | III | 1.8043 | F |
| Pedicularis kansuensis Maxim. | 52.00 | 53.33 | 69.33 | I | 0.7980 | F |
| Pedicularis lachnoglossa Hk. F. | 4.00 | 8.45 | 97.33 | I | 1.5083 | F |
| Pedicularis lasiophrys Maxim. | 2.67 | 0.00 | 0.67 | III | 0.4270 | F |
| Pedicularis lasiophrys Maxim. var. sinica Maxim. | 0.67 | 2.67 | 2.67 | III | 0.3787 | F |
| Pedicularis longiflora Rudolph var. tubiformis （Klotz.） Tsoong | 22.00 | 63.33 | 26.67 | II | 0.6513 | F |
| Pedicularis polyodonta Li | 12.00 | 4.00 | 57.33 | I | 0.5330 | F |
| Pedicularis rudis Maxim. | 0.67 | 12.00 | 2.00 | III | 0.8043 | F |
| Pedicularis semitorta Maxim. | 3.33 | 3.33 | 4.00 | III | 0.4701 | F |
| Pedicularis spicata Pall. | 6.67 | 0.00 | 1.33 | III | 0.5823 | F |
| Pedicularis striata Pall. subsp. arachnoidea (Franch.) Tsoong | 91.33 | 92.67 | 86.00 | IV | 1.0880 | F |
| Pedicularis ternata Maxim. | 0.00 | 0.00 | 0.00 | III | 0.4637 | F |
| Pedicularis tristis L. | 0.67 | 0.67 | 0.00 | III | 0.4557 | F |
| Pedicularis cristatella Pennell et Li | 48.67 | 15.33 | 29.33 | II | 0.6360 | F |
| Peganum multisectum (Maxim.) Bobr. | 76.67 | 88.00 | 86.00 | IV | 5.5997 | F |
| Pennisetum centrasiaticum Tzvel. | 92.67 | 98.00 | 94.00 | IV | 0.4727 | G |
| Pertya discolor Rehd. | 88.33 | 100.00 | 86.67 | IV | 10.1333 | F |
| Phlomis umbrosa Turcz. | 2.67 | 0.00 | 2.67 | III | 3.8657 | F |
| Phragmites australis (Cav.) Trin. ex Steud. | 34.67 | 50.67 | 68.00 | I | 0.0519 | G |
| Phtheirospermum japonicum (Thunb.) Kanitz | 72.67 | 62.00 | 32.67 | II | 0.0933 | F |
| Phytolacca acinosa Roxb. | 0.00 | 14.00 | 4.67 | III | 9.7833 | F |
| Picris hieracioides L. ssp. Japonica Krylv. | 92.67 | 84.33 | 0.67 | II | 1.4297 | F |
| Plantago asiatica L. | 85.33 | 43.33 | 23.33 | II | 0.2053 | F |
| Plantago depressa Willd. | 65.33 | 59.33 | 76.67 | I | 0.2863 | F |
| Pleurospermum cristatum H.de Boiss | 19.33 | 42.00 | 0.00 | II | 2.1780 | F |
| Plumbagella micrantha (Ledeb.) Spach | 21.33 | 29.33 | 14.00 | II | 5.4887 | F |
| Poa annua L. | 45.33 | 36.67 | 79.33 | I | 0.2507 | G |
| Poa attenuata Trin. | 90.67 | 78.67 | 86.00 | IV | 2.3503 | G |
| Poa attenuata Trin. var. vivipara Rendle | 96.00 | 94.18 | 86.00 | IV | 0.1403 | G |
| Poa crymophila Keng ex C.Ling | 95.33 | 94.00 | 78.00 | IV | 0.2060 | G |
| Poa declinata Keng ex L.Liou | 59.33 | 33.33 | 79.33 | I | 0.2653 | G |
| Poa paucifolia Keng | 44.00 | 54.00 | 85.33 | I | 0.1363 | G |
| Poa pratensis L. | 90.00 | 86.67 | 96.67 | IV | 0.1597 | G |
| Poa tunicata Keng | 49.33 | 70.00 | 100.00 | I | 0.2920 | G |
| Polemonium coeruleum L. var. chinense Brand | 25.33 | 50.67 | 73.33 | I | 1.0593 | F |
| Polygonatum verticillatum (L.) All. | 0.00 | 1.33 | 19.33 | I | 10.8133 | F |
| Polygonum fertile (Maxim.) A.J.Li | 92.67 | 96.67 | 0.00 | II | 1.9450 | F |
| Polygonum hydropiper L. | 0.00 | 4.67 | 0.00 | III | 1.0360 | F |
| Polygonum macrophyllum D. Don | 40.00 | 46.00 | 20.00 | II | 1.7060 | F |
| Polygonum orientale L | 8.67 | 0.00 | 0.00 | III | 6.5727 | F |
| Polygonum sibiricum L. | 12.67 | 4.67 | 0.00 | III | 1.2593 | F |
| Polygonum sparsipilosum A.J.L | 1.33 | 2.67 | 15.33 | III | 1.0723 | F |
| Polypogon fugax Nees ex Steud. | 50.00 | 96.67 | 83.33 | I | 1.9033 | G |
| Pomatosace filicula Maxim. | 46.00 | 18.67 | 2.67 | II | 0.6480 | F |
| Potentilla bifurca L. | 0.00 | 51.33 | 0.00 | II | 0.6733 | F |
| Potentilla chinensis Ser. | 17.33 | 49.33 | 6.67 | II | 0.3377 | F |
| Potentilla conferta Bunge | 40.00 | 44.00 | 58.67 | I | 0.3757 | F |
| Potentilla longifolia Willd. ex Schlecht. | 0.00 | 0.00 | 0.00 | III | 0.6230 | F |
| Potentilla multifolia L. | 22.00 | 24.67 | 58.00 | I | 0.3467 | F |
| Potentilla potaninii Wolf | 67.33 | 62.67 | 3.33 | II | 0.2917 | F |
| Potentilla supina L. | 86.67 | 13.33 | 3.33 | II | 0.2917 | F |
| Potentilla tanacetifolia Willd. ex Schlecht. | 30.67 | 60.00 | 38.67 | II | 0.4683 | F |
| Prenanthes macrophylla Franch. | 8.00 | 42.00 | 4.67 | II | 0.4443 | F |
| Prenanthes tatarinowii Maxim. | 0.00 | 6.67 | 33.33 | I | 0.7380 | F |
| Primula gemmifera Batal. | 26.67 | 4.00 | 2.00 | II | 0.0410 | F |
| Primula nutans Georgi | 63.33 | 50.00 | 10.00 | II | 0.0620 | F |
| Primula orbicularis Hemsl. | 3.33 | 0.00 | 0.00 | III | 0.2370 | F |
| Primula tangutica Duthie | 0.00 | 0.00 | 10.00 | III | 0.0860 | F |
| Ptilagrostis concinna (Hook. f.) Roshev. | 89.33 | 94.67 | 94.67 | IV | 0.6180 | G |
| Ptilagrostis dichotoma Keng ex Tzvel. | 84.67 | 88.00 | 100.00 | IV | 0.8053 | G |
| Pycreus sanguinolentus (Vahl) Nees | 10.67 | 9.33 | 0.67 | III | 0.2523 | G |
| Pyrethrum tatsienense (Bur. et Franch.) Ling ex Shih | 80.00 | 90.67 | 98.00 | IV | 0.4743 | F |
| Ranunculus tanguticus (Maxim.) Ovcz. | 13.33 | 4.67 | 0.00 | III | 0.4107 | F |
| Rhodiola dumulosa (Franch.) S.H.Fu | 54.67 | 77.33 | 20.67 | II | 0.1567 | F |
| Rhodiola eurycarpa (Fröd.) S.H.Fu | 0.67 | 0.00 | 38.67 | I | 0.0443 | F |
| Rhodiola kirilowii (Regel) Maxim. | 60.00 | 70.67 | 98.00 | I | 0.0800 | F |
| Rodgersia aesculifolia Batal. | 86.00 | 19.33 | 46.67 | II | 0.0623 | F |
| Roegneria breviglumis Keng | 84.44 | 97.78 | 96.00 | IV | 3.4680 | G |
| Roegneria dura (Keng) Keng | 52.00 | 76.67 | 18.67 | II | 4.0263 | G |
| Roegneria kokonorica Keng | 56.00 | 60.00 | 91.67 | I | 3.4463 | G |
| Roegneria stricta Keng | 96.00 | 97.33 | 22.00 | II | 3.4133 | G |
| Roegneria varia Keng et S.L.Cheng | 86.00 | 96.00 | 99.33 | IV | 2.7773 | G |
| Rorippa palustris (L.) Bess. | 88.67 | 13.33 | 25.33 | II | 0.0593 | F |
| Rumex crispus L. | 96.67 | 74.67 | 88.00 | IV | 1.6020 | F |
| Rumex nepalensis Spreng. | 98.00 | 100.00 | 100.00 | IV | 2.8777 | F |
| Rumex patientia L. | 88.67 | 73.33 | 82.00 | IV | 1.6443 | F |
| Rumex pseudonatronatus (Borb.) Borb. ex Murb. | 16.00 | 7.33 | 37.33 | I | 2.0653 | F |
| Salvia przewalskii Maxim. | 21.33 | 31.33 | 66.67 | I | 3.9960 | F |
| Salvia roborowskii Maxim. | 15.33 | 16.67 | 9.33 | III | 3.0297 | F |
| Sambucus chinensis Lindl. | 0.00 | 0.00 | 1.33 | III | 0.7953 | F |
| Sanguisorba officinalis L. | 34.00 | 16.67 | 5.00 | II | 2.4583 | F |
| Saussurea globosa Chen | 50.00 | 14.00 | 45.33 | I | 1.8473 | F |
| Saussurea hieracioides Hook.f. | 80.00 | 94.00 | 85.33 | IV | 2.1340 | F |
| Saussurea iodostegia Hance | 92.67 | 82.67 | 73.33 | II | 1.4207 | F |
| Saussurea japonica (Thunb.) DC. | 87.33 | 87.33 | 91.33 | IV | 1.1217 | F |
| Saussurea macrota Franch. | 74.00 | 86.00 | 68.67 | II | 1.3800 | F |
| Saussurea parviflora (Poir.) DC. | 37.33 | 68.00 | 73.33 | I | 1.3913 | F |
| Saussurea stella Maxim. | 100.00 | 48.67 | 96.67 | I | 1.2900 | F |
| Saussurea sylvatica Maxim. | 36.00 | 41.33 | 56.67 | I | 2.2937 | F |
| Saussurea variiloba Ling | 38.67 | 86.67 | 39.33 | II | 1.8473 | F |
| Saxifraga egregia Engl. | 9.33 | 4.00 | 4.67 | III | 0.0210 | F |
| Saxifraga montana H. Smith | 17.33 | 9.33 | 0.67 | III | 0.0383 | F |
| Schizonepeta multifida (L.) Briq. | 74.00 | 86.00 | 85.33 | IV | 0.3090 | F |
| Scirpus distigmaticus (Kükenth.) Tang et Wang | 0.00 | 0.00 | 0.00 | III | 0.6683 | G |
| Scorzonera austriaca Willd. | 55.56 | 24.44 | 72.73 | I | 5.4260 | F |
| Scrofella chinensis Maxim. | 67.33 | 56.67 | 44.67 | II | 0.0597 | F |
| Scrophularia incisa Weinm. | 2.67 | 2.00 | 1.33 | III | 0.3407 | F |
| Scutellaria baicalensis Georgi | 42.00 | 20.00 | 25.00 | II | 1.5490 | F |
| Sedum aizoon L. | 94.00 | 93.00 | 50.67 | II | 0.0643 | F |
| Senecio argunensis Turcz. | 58.00 | 79.33 | 94.00 | I | 0.5473 | F |
| Senecio densiserratus Chang | 55.33 | 92.67 | 87.33 | I | 0.8110 | F |
| Senecio dubitabilis C.Jeffrey et Y.L.Chen | 98.00 | 97.33 | 98.67 | IV | 0.1943 | F |
| Seseli squarrulosum Shan et Sheh | 0.00 | 60.00 | 93.33 | I | 0.8190 | F |
| Setaria glauca (L.) Beauv. | 2.67 | 2.00 | 87.33 | I | 0.3807 | G |
| Setaria viridis (L.) Beauv. | 1.33 | 0.00 | 1.33 | III | 0.7077 | G |
| Sibbaldia procumbens L. var. aphanopetala (Hand.-Mazz.) Yü et Li | 62.00 | 36.00 | 13.33 | II | 0.9170 | F |
| Siegesbeckia pubescens Makino | 78.00 | 91.33 | 32.00 | II | 2.8503 | F |
| Silene conoidea L. | 57.33 | 99.00 | 30.30 | II | 1.2483 | F |
| Silene fortunei Vis | 96.67 | 96.67 | 85.33 | IV | 0.5267 | F |
| Silene gracilicaulis C. L. Tang | 80.00 | 100.00 | 99.33 | IV | 0.3443 | F |
| Silene pterosperma Maxim. | 73.33 | 96.00 | 32.00 | II | 0.2637 | F |
| Silene repens Patr. | 90.67 | 71.33 | 89.33 | IV | 0.2107 | F |
| Sinochasea trigyna Keng | 59.33 | 81.33 | 86.67 | I | 1.8803 | G |
| Sinopodophyllum hexandrum (Royle) Ying | 0.00 | 12.67 | 6.48 | III | 27.3323 | F |
| Sinosenecio euosmus (Hand.-Mazz.) B. Nord. | 36.67 | 27.33 | 96.67 | I | 0.2598 | F |
| Sisymbrium heteromallum C.A.Mey. | 5.33 | 10.00 | 9.33 | III | 0.2730 | F |
| Solanum alatum Moench | 46.00 | 42.00 | 97.33 | I | 2.1303 | F |
| Sorosers erysimoides (Hand-Mazz) Shih. | 66.67 | 12.00 | 30.00 | I | 1.0833 | F |
| Souliea vaginata (Maxim.) Franch. | 0.00 | 0.00 | 0.00 | III | 2.3220 | F |
| Sphallerocarpus gracilis (Trevir) K.-Pol. | 8.00 | 8.00 | 2.00 | III | 3.6413 | F |
| Stachys sieboldi Miq. | 0.67 | 0.00 | 8.67 | I | 2.2083 | F |
| Stellaria dianthifolia Williams | 66.00 | 90.00 | 95.33 | I | 0.2290 | F |
| Stellaria graminea L. | 90.67 | 100.00 | 94.00 | IV | 0.2290 | F |
| Stellaria neglecta Weihe ex Fingerh. | 94.67 | 82.67 | 74.00 | II | 0.2383 | F |
| Stellaria parviumbellata Y. Z. Zhao | 29.52 | 22.00 | 1.33 | II | 0.2383 | F |
| Stellera chamaejasme L. | 0.00 | 3.33 | 2.00 | III | 1.5483 | F |
| Stephanachne nigrescens Keng | 81.33 | 91.33 | 13.33 | II | 2.0907 | G |
| Stipa bungeana Trin. | 24.44 | 82.22 | 27.33 | II | 1.0640 | G |
| Stipa przewalskyi Roshev. | 72.00 | 73.33 | 66.00 | II | 4.5120 | G |
| Swertia bifolia Batal. | 9.33 | 4.00 | 0.67 | III | 0.1463 | F |
| Swertia bimaculata (Sieb.et Zucc.)Hook.f.et Thoms. ex Clarke | 67.33 | 25.33 | 0.00 | II | 0.1057 | F |
| Swertia diluta (Turcz.) Benth. et Hook. f. | 10.67 | 1.33 | 60.00 | I | 0.2000 | F |
| Swertia erythrosticta Maxim. | 44.67 | 35.33 | 16.67 | II | 0.0707 | F |
| Swertia franchetiana H. Smith | 51.33 | 15.00 | 1.00 | II | 0.1463 | F |
| Swertia tetraptera Maxim. | 0.67 | 1.33 | 0.00 | III | 0.6497 | F |
| Taraxacum calanthodium Dahlst. | 77.78 | 90.00 | 70.00 | II | 0.4483 | F |
| Taraxacum maurocarpum Dahlst. | 88.67 | 82.00 | 55.33 | II | 0.9270 | F |
| Taraxacum mongolicum Hand.-Mazz. | 85.33 | 88.18 | 1.33 | II | 0.3987 | F |
| Thalictrum alpinum L. var. elatum Ulbr. | 28.33 | 66.67 | 88.67 | I | 0.8593 | F |
| Thalictrum macrorhynchum Franch. | 0.00 | 0.00 | 0.00 | III | 4.5233 | F |
| Thalictrum minus L. | 85.33 | 89.33 | 64.67 | II | 1.4697 | F |
| Thalictrum minus L. var. hypoleucum (Sieb.et Zucc.) Miq. | 93.33 | 85.13 | 48.67 | II | 3.3987 | F |
| Thalictrum przewalskii Maxim. | 89.33 | 78.67 | 34.67 | II | 1.3460 | F |
| Thalictrum rutifolium Hook.f. et Thoms. | 46.67 | 4.00 | 48.89 | I | 1.5843 | F |
| Thalictrum uncatum Maxim. | 81.33 | 66.67 | 6.67 | II | 3.9677 | F |
| Thermopsis lanceolata R. Br. | 17.33 | 18.67 | 10.67 | III | 24.9017 | F |
| Thesium refractum C.A.Mey. | 0.00 | 8.33 | 3.33 | III | 5.0890 | F |
| Thymus mongolicus Ronn. | 25.33 | 59.33 | 24.67 | II | 0.2237 | F |
| Tibetia himalaica (Baker) Tsui | 14.67 | 18.67 | 58.00 | I | 2.0780 | F |
| Tongoloa elata Wolff | 7.33 | 2.00 | 0.00 | III | 0.5273 | F |
| Tragus berteronianus Schult. | 49.33 | 58.67 | 60.67 | I | 0.2433 | G |
| Triglochin palustre L. | 94.73 | 91.33 | 67.33 | II | 0.5830 | F |
| Trisetum clarkei (Hook. f.) R.R.Stewart | 47.33 | 60.67 | 94.67 | I | 0.2423 | G |
| Vaccaria segetalis (Neck.) Garcke | 100.00 | 100.00 | 96.00 | IV | 3.1660 | F |
| Valeriana officinalis L. | 15.33 | 3.33 | 0.00 | II | 0.6643 | F |
| Verbena officinalis L. | 51.33 | 3.33 | 9.33 | II | 0.3457 | F |
| Veronica anagallisaquatica L. | 96.67 | 10.00 | 4.67 | II | 0.0227 | F |
| Veronica ciliata Fisch. | 49.33 | 28.00 | 35.33 | II | 0.0493 | F |
| Veronica eriogyne H.Winkl. | 32.67 | 66.00 | 12.67 | II | 0.0540 | F |
| Veronica rockii H.L.Li | 38.00 | 69.33 | 4.00 | II | 0.0263 | F |
| Veronica szechuanica Batal. | 4.00 | 0.00 | 0.00 | III | 0.4293 | F |
| Vicia angustifolia L. ex Reichard | 5.33 | 2.67 | 21.33 | I | 16.3960 | F |
| Vicia cracca L. | 0.00 | 2.67 | 4.76 | III | 9.5683 | F |
| Vicia multicaulis Ledeb. | 11.33 | 8.00 | 6.67 | III | 8.6033 | F |
| Vicia unijuga A.Br. | 10.00 | 5.33 | 10.00 | III | 6.4190 | F |
| Xanthopappus subacaulis C. Winkl. | 75.33 | 88.24 | 91.33 | IV | 3.7953 | F |
| Zygophyllum mucronatum Maxim. | 0.00 | 3.33 | 14.67 | III | 2.6790 | F |
