# Supplementary material for: Effect of Diurnal Fluctuating versus Constant Temperatures on Germination of 445 Species from the Eastern Tibet Plateau
Source: PLoS One. 2013 Jul 24;8(7):e69364. doi: 10.1371/journal.pone.0069364 (PMC3722265; doi:10.1371/journal.pone.0069364)
Supplement: Table S3 — Daily temperature fluctuation on the eastern Tibet Plateau (Maqu) and three other places with the similar latitude but different altitudes. (DOC) [file pone.0069364.s003.doc]

**Table S3**. Daily temperature fluctuation on the eastern Tibet Plateau (Maqu) and three other places with the similar latitude but different altitudes.

| Location | Altitude (m) | J | F | M | A | M | J | J | A | S | O | N | D | Mean |
| --- | --- | --- | --- | --- | --- | --- | --- | --- | --- | --- | --- | --- | --- | --- |
| Maqu; 33°57'N, 102°04'E | 3500 | 17.4 | 19.1 | 14.9 | 16 | 14 | 11.6 | 11.6 | 13 | 14.2 | 12.2 | 17.5 | 18.8 | 15 |
| Lanzhou; 36°01'N, 103°59'E | 1508 | 14.5 | 16.1 | 13.4 | 13.9 | 14.4 | 13.3 | 12.8 | 11.1 | 10.6 | 12.8 | 13.3 | 13.9 | 13.3 |
| Xi’an; 34°15'N, 108°35'E | 412 | 9.4 | 9.5 | 11.1 | 11.7 | 10.5 | 10.5 | 8.3 | 8.4 | 8.4 | 8.4 | 10 | 9.4 | 9.59 |
| Qingdao; 36°04'N, 120°19'E | 77 | 6.6 | 6.6 | 7.7 | 7.7 | 7.2 | 6.6 | 5 | 5.5 | 7.2 | 7.8 | 7.8 | 6.6 | 6.86 |
